# Immunotherapy of cytomegalovirus infection by low-dose adoptive transfer of antiviral CD8 T cells relies on substantial post-transfer expansion of central memory cells but not effector-memory cells

**DOI:** 10.1371/journal.ppat.1011643

**Published:** 2023-11-16

**Authors:** Rafaela Holtappels, Sara Becker, Sara Hamdan, Kirsten Freitag, Jürgen Podlech, Niels A. Lemmermann, Matthias J. Reddehase

**Affiliations:** 1 Institute for Virology, University Medical Center of the Johannes Gutenberg-University Mainz, Mainz, Germany; 2 Research Center for Immunotherapy (FZI), University Medical Center of the Johannes Gutenberg-University Mainz, Mainz, Germany; 3 Institute of Virology, Medical Faculty, University of Bonn, Bonn, Germany; University of Wisconsin-Madison, UNITED STATES

## Abstract

Cytomegaloviruses (CMVs) are host species-specific in their replication. It is a hallmark of all CMVs that productive primary infection is controlled by concerted innate and adaptive immune responses in the immunocompetent host. As a result, the infection usually passes without overt clinical symptoms and develops into latent infection, referred to as “latency”. During latency, the virus is maintained in a non-replicative state from which it can reactivate to productive infection under conditions of waning immune surveillance. In contrast, infection of an immunocompromised host causes CMV disease with viral multiple-organ histopathology resulting in organ failure. Primary or reactivated CMV infection of hematopoietic cell transplantation (HCT) recipients in a “window of risk” between therapeutic hemato-ablative leukemia therapy and immune system reconstitution remains a clinical challenge. Studies in the mouse model of experimental HCT and infection with murine CMV (mCMV), followed by clinical trials in HCT patients with human CMV (hCMV) reactivation, have revealed a protective function of virus-specific CD8 T cells upon adoptive cell transfer (AT). Memory CD8 T cells derived from latently infected hosts are a favored source for immunotherapy by AT. Strikingly low numbers of these cells were found to prevent CMV disease, suggesting either an immediate effector function of few transferred cells or a clonal expansion generating high numbers of effector cells. In the murine model, the memory population consists of resting central memory T cells (TCM), as well as of conventional effector-memory T cells (cTEM) and inflationary effector-memory T cells (iTEM). iTEM increase in numbers over time in the latently infected host, a phenomenon known as ‘memory inflation’ (MI). They thus appeared to be a promising source for use in immunotherapy. However, we show here that iTEM contribute little to the control of infection after AT, which relies almost entirely on superior proliferative potential of TCM.

## Author summary

Immunotherapy of reactivated cytomegalovirus (CMV) infection in immunocompromised HCT recipients by adoptive transfer (AT) of antiviral CD8 T cells is the last resort to fight virus variants that have acquired resistance to standard antiviral drugs. Provision of cell numbers high enough for clearance of productive infection remains a logistical limitation for AT to become clinical routine. Although use of donor memory CD8 T cells has become the standard in clinical AT, little is known about the relative antiviral efficacies of memory CD8 T-cell activation subsets, such as central memory cells (TCM) and different populations of effector-memory cells (TEM). A reliable quantitative comparison of the antiviral efficacies of memory CD8 T-cell subsets is precluded in clinical investigation, because independent cohorts of AT donors and recipients necessarily differ in many genetic, immunologic, and virologic variables. Therefore, this is a question for which a preclinical animal model is predestined. We show here in the well-established mouse model of low-dose AT that CMV infection is by far most efficiently controlled by virus-specific TCM, due to their superior potential to proliferate even in extra-lymphoid tissue preventing intra-tissue virus spread. For clinical AT, our data provide an argument to favor transfer of TCM rather than TEM.

## Introduction

The clinical relevance of human cytomegalovirus (hCMV), the prototype member of the β-subfamily of the herpes virus family [1], results from severe and often lethal CMV disease that it causes under conditions of compromised immunity [2–4]. A concern with significant health system impact are birth defects resulting from congenital infection of immunologically immature fetuses, a syndrome historically known as cytomegalic inclusion disease (CID) (for overviews, see [5,6]). Risk groups of medical and logistic challenge in transplantation centers worldwide are iatrogenically immunocompromised recipients of hematopoietic cell transplantation (HCT) [7] and of solid organ transplantation (SOT) [8,9]. In those patients, hCMV reactivation within a latently infected transplant or within latently infected organs of the recipient causes graft failure and multiple organ disease, with interstitial pneumonia representing the most threatening clinical manifestation specifically in HCT (for clinical reviews, see [10–13]).

As CMVs are host-species specific in their replication based on host range determinants [14–16], understanding of the underlying mechanisms of CMV disease and immune control of infection comes at its limits when research is restricted to clinical investigation. In particular, human genetics cannot be manipulated, and hCMV mutants cannot be used experimentally for designed *in vivo* studies aimed at identifying the roles of host and viral genes involved in pathogenesis and immunity. Of all animal models of CMV disease and infection control, infection of the mouse with murine CMV (mCMV) is the most advanced with respect to host genetics [17]. As hCMV and mCMV differ genetically, each not just containing homologous genes with related functions but also “private genes” co-evolved with and thereby adapted to the respective host species [1,18–20], the results for mCMV cannot be translated par for par to hCMV.

Nonetheless, the mouse model has identified basic principles common to all CMVs [21], and is an acknowledged model of value for understanding clinical CMV disease and immune control, as well as for developing strategies of antiviral immunotherapy in HCT patients [22]. As paramount examples for successful clinical translation, mouse models of experimental syngeneic [23–25] and allogeneic [26,27] HCT (for overviews see [28,29]), and of control of infection by adoptive transfer (AT) of virus-specific CD8 T cells [30–34] were of predictive value for the management of human infection. Specifically, results from mouse models have paved the way to “pre-emptive immunotherapy” of hCMV infection in HCT recipients. This means prevention of CMV disease by transfer of virus-specific CD8 T cells as soon as virus reactivation is detected in the routine follow-up screening long before clinical diagnosis of CMV disease manifestations [35–39]. Although AT in a clinical scale is still logistically demanding, it is the last resort to combat infections by virus variants that have acquired resistance to standard antiviral drugs and is generally an option to avoid drug toxicity [40–46].

The source of CD8 T cells for clinical immunotherapy are mostly latently infected but otherwise healthy donors who bear hCMV-specific memory CD8 T cells resulting from previous infection. Ideally, the CD8 T-cell donor is the one who has been HLA class-I matched for the HCT, so that the viral epitope-specificity of the memory CD8 T cells matches with the epitopes presented by the HLA class-I molecules of the combined HCT and AT recipient. Studies conducted on mice ([47–49], for a review see [50]), as well as clinical trials [38,39,51], have consistently demonstrated that memory CD8 T cells are more effective in immunotherapy when compared to terminally-differentiated effector CD8 T cells of *in vitro*-propagated cytolytic T-cell lines (CTLL) of identical epitope-specificity. Accordingly, the previous approach of propagating CD8 T cells in cell cultures to achieve sufficiently high cell numbers for AT [36,52] is no longer being pursued. Suspected reasons were loss of functional avidity in the recognition of presented antigenic peptides or a loss of *in vivo* homing properties as a result of selection during expansion in cell culture.

In latently infected mice, the memory CD8 T-cell population consists of three major subpopulations, all expressing CD44 in distinction to naïve, unprimed CD8 T cells: central memory T cells (TCM) characterized by the cell surface phenotype CD62L^+^KLRG1^-^, as well as conventional T effector-memory cells (cTEM) and inflationary T effector-memory cells (iTEM) with the cell surface phenotypes CD62L^-^KLRG1^-^ and CD62L^-^KLRG1^+^, respectively [53]. iTEM were previously referred to as short-lived effector cells (SLEC) [54] but were then shown to differ from terminally-differentiated effector T cells (TEC) by dependence on IL-15 [55]. After high-dose systemic but not after low-dose local infection [53], the iTEM population reflects a phenomenon named “memory inflation” (MI), as numbers of iTEM increase almost steadily over time during latent mCMV infection (for reviews, see [56–59]). It is current view that the latent viral genome is not transcriptionally silent but that sporadic episodes of limited viral gene expression [60–62], proposed to take place in latently infected endothelial cells [62–64] and likely also in latently infected PDGFRα^+^ fibroblasts [65], lead to the presentation of antigenic peptides that drive MI [56].

Here we have sorted TCM, cTEM, and iTEM for AT into immunocompromised and infected recipients to directly quantify their individual contributions to the control of infection. Notably, prevention of viral spread and pathogenesis was based almost entirely on TCM, and correlated with the proliferative capacity, which was highest for TCM and lowest for iTEM. In conclusion, efficient expansion of antiviral TCM within the host is crucial for control of infection and prevention of CMV disease upon pre-emptive immunotherapy by AT.

## Results

### Framework conditions of the experimental AT mouse model

As reviewed by Moss and Rickinson [66], AT is a promising immunotherapy of viral infections in HCT recipients, also beyond CMV. It is long established in the murine model system, as well as by clinical trials, that lethal CMV infections of the immunocompromised host can be prevented by AT of CMV-specific CD8 T cells, provided that the protective cells are administered early after infection [35–39]. This is the basis for initiating clinical pre-emptive AT immunotherapy of hCMV reactivation in HCT recipients as soon as it is detected by sensitive routine follow-up monitoring, a clinical regimen originally established for antiviral drug therapy [7].

Clinical AT in HCT patients is highly individualized. Treatment regimens depend on the preexisting hematopoietic malignancy and the degree of therapeutic bone marrow ablation of the recipient, the stem cell source and cellular composition of the graft (T-cell-depleted or non-depleted), the CMV status of both donor and recipient, and the need for immunosuppressive prevention of graft-versus-host disease (GvHD) in the presence of immunogenetic differences. Other variables that are difficult to manage clinically include often underestimated differences in host cell tropism, pathogenicity, and immunogenicity of the CMV strains involved (for more recent reviews, see [67–69]), and the unpredictable time of CMV reactivation in individual HCT recipients. With this in mind, no experimental model can cover all clinical cases, and all models are necessarily highly reductionist. This insight makes it all the more important to define the specific question addressed and the possibilities and limitations of the model used.

**Fig 1:**
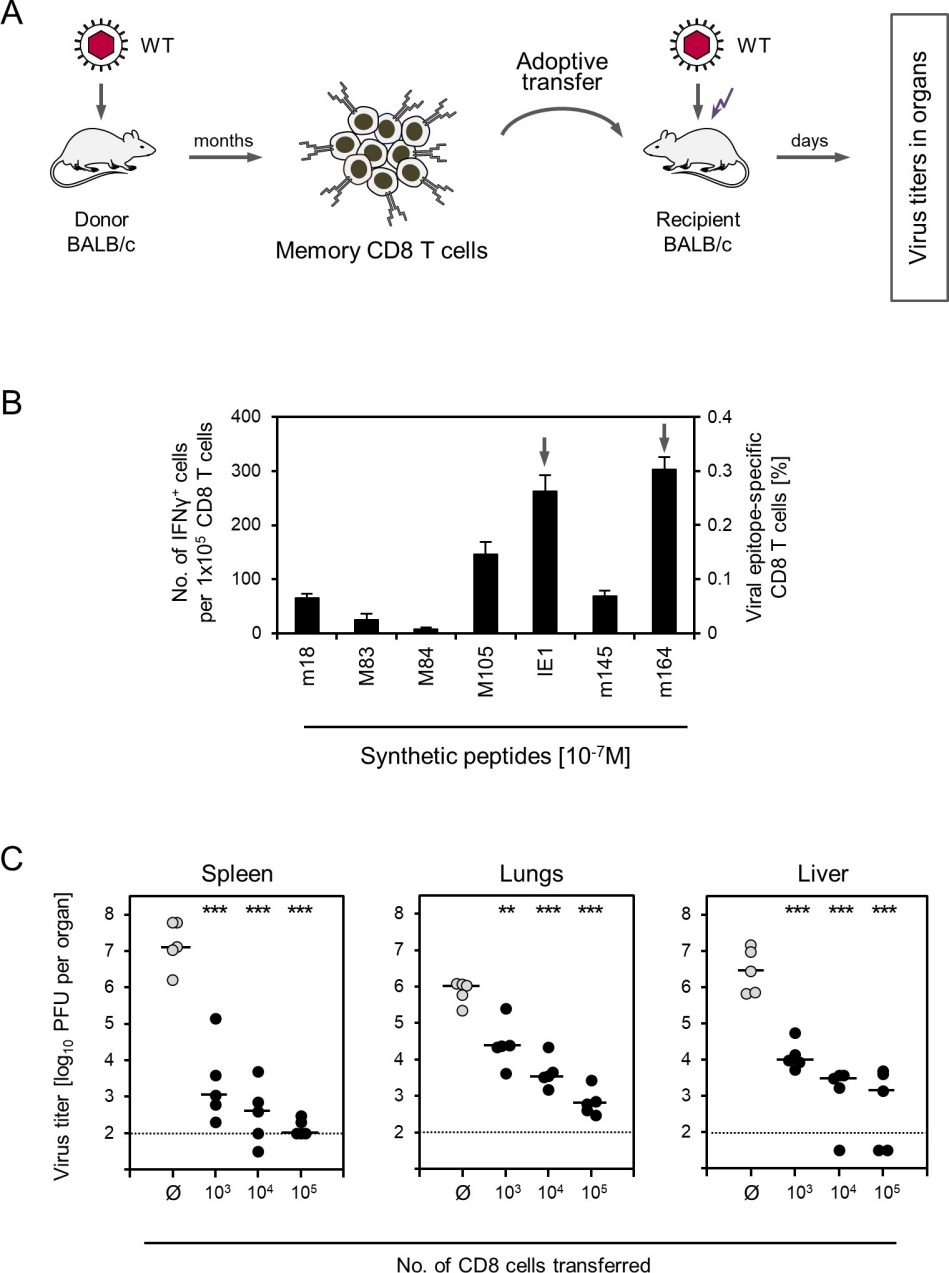
Control of infection by AT of memory CD8 T cells. (A) Sketch of the experimental protocol. Total memory CD8 T cells derived from latently infected BALB/c mice as AT donors are transferred into BALB/c AT recipient mice that were immunocompromised by γ-irradiation (flash symbol) and infected with mCMV-WT (WT). See the body of the text for a detailed explanation of the model. (B) Frequencies of memory CD8 T cells specific for the viral epitopes indicated. Bars represent the frequencies of cells stimulated by viral antigenic peptides to secrete IFNγ in an ELISpot assay, determined by intercept-free linear regression analysis. Error bars represent the 95% confidence intervals. Arrows highlight the frequencies of the two immunodominant CD8 T-cell specificities IE1 and m164. (C) Virus titers in spleen, lungs, and liver were determined on day 11 after AT of graded cell numbers indicated. Ø, no AT performed. Symbols represent data from individual mice (n = 5 per group). Median values are marked. Dotted lines represent the detection limit of the virus plaque assay. (PFU) plaque-forming units. Asterisk-coded statistical significance levels for differences between the AT groups and the no-AT control group (Ø): (**) P < 0.01 and (***) P < 0.001.

The specific aim of our study was to predict the fate and antiviral efficacies of transferred donor-derived CMV-specific memory CD8 T cells and their activation subsets in immunocompromised and CMV-infected, potential HCT recipients in the well-defined mouse model of syngeneic AT (reviewed in [50,70]). In this model (Fig 1A), immunocompetent BALB/c mice intended to serve as AT donors are immunized by a local intra-plantar acute infection that is rapidly cleared and develops into a latent infection associated with the establishment of immunologic memory [53]. At times usually beyond 2 months, memory CD8 T cells isolated from the spleens of the latently infected donor mice are transferred intravenously into BALB/c recipient mice that have been hemato-ablated by whole-body γ-irradiation. HCT was not performed to avoid masking the effects of AT by endogenous CD8 T-cell reconstitution that would otherwise result from HCT (reviewed in [28,71]).

In HCT patients, hCMV reactivation can occur at any time after and even before HCT, but is usually observed between 3 and 5 weeks after HCT [7]. In latently infected mice, virus reactivation after hemato-ablative treatment is a stochastic event with an incidence defined by the load of latent viral genomes. As a consequence, within a test cohort of latently infected mice, reactivation occurs at different times and in different organs, and not necessarily in all individual mice [72,73]. These systematic variables exclude reliable cohort comparisons of antiviral control by AT of different memory CD8 T-cell subsets. The current model thus uses primary infection as a “surrogate for reactivation” taking place at the same time and in all individual mice of experimental AT test cohorts. Despite this adapted protocol, a clinical time frame of reactivation between 3 and 5 weeks after HCT cannot be reproduced, because even uninfected recipient mice would die from the third week after hemato-ablation if they did not receive HCT, as well as after low-dose HCT [28,30].

With this rationale, the model is built to reflect a very early hCMV reactivation prior to hematopoietic reconstitution of antiviral CD8 T cells by HCT. This is technically achieved by infecting the AT recipient mice with mCMV shortly after hemato-ablation and with no HCT being performed, followed by pre-emptive experimental AT.

### Adoptive transfer of remarkably low numbers of virus-specific memory CD8 T cells controls virus replication at multiple organ sites

The term “memory cells” is here used collectively for a mixture of antigen-experienced cells in different stages of differentiation and activation, comprising resting TCM as well as activated cTEM and iTEM [53]. In order to calibrate the system, we first studied the unseparated memory CD8 T-cell population. Cytofluorometric (CFM) analysis revealed presence of the three main memory T-cell populations defined by the expression of CD62L and KLRG1, that is, the TCM, cTEM, and iTEM (S1A Fig). Almost all cells of the memory population expressed CD44, distinguishing them from CD44^-^ naïve cells that have not yet encountered antigen and represent a minority of peripheral CD8 T cells. As noted in the important work by Welsh and Selin, “no one is naïve” [74], so that memory cells of numerous antigen specificities make up the memory cell population. Trivially, most memory CD8 T cells are not specific for the virus under investigation but collectively reflect all preceding antigen encounters in the past life.

To determine the virus-specific fraction of memory CD8 T cells, total CD8 cells derived from the spleen of latently infected BALB/c mice were enriched by positive immunomagnetic cell sorting (S1B Fig) and tested by an IFNγ-based ELISpot-Assay covering all response-relevant CD8 T-cell epitopes of mCMV in the *H-2*^*d*^ haplotype (for a list of peptide sequences and the presenting MHC class-I molecules, see [50]) (Fig 1B). In accordance with previous data [25,75], memory CD8 T cells specific for the epitopes IE1 and m164 dominated the response, and cells specific for the tested panel of epitopes added up to ~0,9% of the population. Other already identified epitopes, which were not included in the analysis, are known to generally contribute little to the overall CD8 T-cell response, and previous work, using a viral genome-wide open reading frame (ORF) expression library [76], did not reveal the existence of unidentified CD8 T-cell epitopes that would make a notable contribution to the memory response [77]. It is therefore reasonable to conservatively extrapolate that mCMV epitope-specific memory cells accounted for not more than ~1% of the CD8 T-cell population. Given the universe of antigens, this is nevertheless a remarkable allocation of the memory CD8 T-cell pool to a single viral pathogen.

The thus characterized cells were then used as donor cells for syngeneic AT into infected immunocompromised BALB/c recipients (recall Fig 1A). As shown by previous work in this model [30,78], control recipients left without AT start dying from day 12 onward of CMV disease characterized by extensive cytopathogenic viral spread leading to tissue lesions in vital organs. This defined the read-out day 11 in our AT experiments. Notably, as few as 1,000 transferred cells, containing just ~10 viral epitope-specific cells (Fig 1B), significantly reduced viral replication in three organs tested, namely in spleen, lungs, and liver. Almost clearance of the infection was achieved by AT of 100,000 cells, corresponding to ~1,000 viral epitope-specific cells (Fig 1C).

### Control of infection is mediated by viral epitope-specific memory CD8 T cells

We have previously reported a strategy to test for viral epitope-specificity of CD8 T-cell priming, target cell recognition, and protection against *in vivo* virus replication in AT models by site-directed virus mutagenesis of the C-terminal amino acid residue that anchors an antigenic peptide to the presenting MHC class-I molecule [79]. This strategy was first applied to the deletion of IE1 peptide antigenicity and immunogenicity in mCMV mutant IE1-L176A, in which the MHC class-I (L^d^) anchor residue Leu at the C-terminal position of the antigenic IE1 peptide is replaced with Ala [61]. More recently, this strategy was employed to show viral epitope-specificity of antiviral protection upon AT in a mouse model of “humanized antigen presentation” using TCR-transduced murine or human CD8^+^ CTLL specific for an antigenic peptide of hCMV presented on tissue cells of HLA-A2 transgenic mice [32]. Notably, AT of these CTLL, both murine and human CTLL, into recipients infected with recombinant mCMV expressing the authentic antigenic peptide led to tissue infiltration by CD8 T cells, associated with the formation of “nodular inflammatory foci (NIF)” to which infection is confined and eventually cleared. In contrast, when AT recipients were infected with recombinant mCMV expressing the C-terminal Ala-variant of the peptide, infiltrates were missing almost completely and the virus was spreading unhindered with consequent viral histopathological lesions [32].

**Fig 2:**
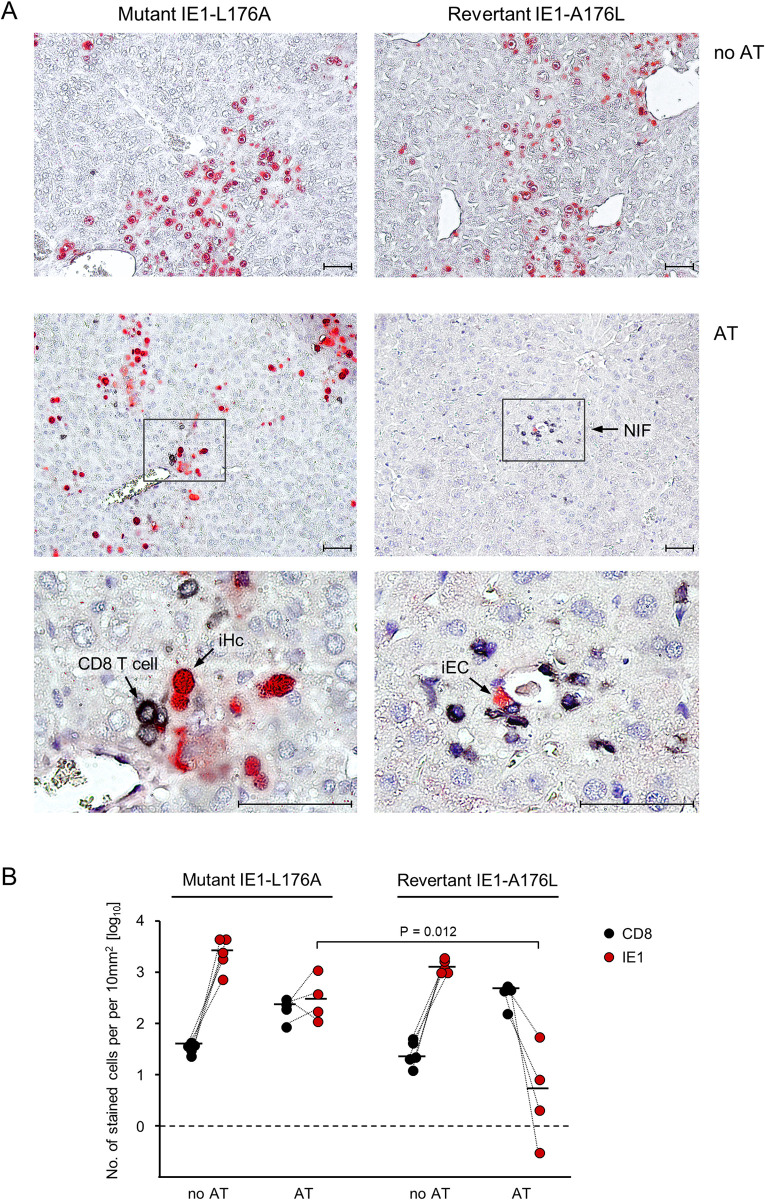
Viral epitope-specificity of antiviral control. AT was performed with 10^4^ IE1-epitope specific memory CD8 T cells isolated from splenocytes of latently-infected BALB/c donor mice by negative immunomagnetic pre-enrichment of CD8^+^ cells followed by fluorescence-based sorting of cells expressing IE1 epitope-specific TCRs. AT recipients were infected either with the IE1 epitope mutant virus mCMV-IE1-L176A or the corresponding revertant virus mCMV-IE1-A176L. Analyses were performed on day 11 after AT and infection. (A) 2C-IHC images of liver tissue sections. (Left panels) AT recipients infected with mCMV-IE1-L176A. (Right panels) AT recipients infected with mCMV-IE1-A176L. Infected liver cells are identified by red staining of the intra-nuclear viral protein IE1. (iHc) infected hepatocyte. (iEC) infected capillary endothelial cell. Tissue-infiltrating IE1-specific CD8 T cells are identified by black staining of the CD8a molecule. Light hematoxylin counterstaining reveals the context of liver tissue. (Upper panel, no AT) Unhindered intra-tissue spread of both viruses. (Center and lower panels, AT) The center panel provides a low-magnification overview, showing the confinement of infection by formation of nodular inflammatory foci (NIF) only after infection with revertant virus mCMV-IE1-A176L expressing the antigenic IE1 peptide on infected cells. Frames demarcate regions of interest resolved to greater detail in the lower panel images. Bar markers represent 50μm. (B) Quantitation of infected IE1^+^ liver cells (red dots) and tissue-infiltrating CD8 T cells (black dots) in representative 10-mm^2^ liver tissue section areas. Symbols represent cell counts for AT recipients (n = 4–5 per group) tested individually. Linked data are connected by dotted lines, median values are marked. The dashed line indicates the detection limit of the assay.

Here we tested if epitope-specificity of antiviral protection also applies to *ex vivo* sorted IE1 epitope-specific memory cells that differ from CTLL in activation stage, proliferative capacity, and tissue homing properties. For this, immunocompromised AT recipient mice were infected either with the mCMV mutant IE1-L176A, in which presentation of the IE1 epitope is largely reduced, or with the corresponding revertant IE1-A176L (Fig 2). When no AT was performed, two-color immunohistochemical (2C-IHC) analysis of representative liver tissue sections, which were stained for the CD8 molecule and the viral IE1 protein, revealed absence of CD8 T cells and comparable tissue spread of both viruses. Viral spread becomes visible as extended tissue areas with infected IE1^+^ liver cells, which are predominantly hepatocytes (iHc) and endothelial cells (iEC) [80,81]. When AT was performed, CD8 T cells infiltrating liver tissue infected with the mutant virus were found in proximity to infected cells, but controlled infection inefficiently. In contrast, after infection with the revertant virus, CD8 T cells clustered more densely around infected cells to form NIF, thereby confining and eventually resolving the infection (Fig 2A).

Quantitation of liver tissue-infiltrating CD8 T cells and of infected liver cells in the respective groups of mice confirmed a significantly better control of the revertant virus expressing the authentic antigenic IE1 peptide that binds with high affinity to the presenting MHC class-I molecule (Fig 2B). Some tissue infiltration by CD8 T cells and partial control of mutant virus IE1-L176A does not necessarily indicate a contribution of epitope-unspecific cells. It is more likely explained by low-affinity MHC class-I binding of the L176A peptide [61,82] sufficient for presenting the minimal antigenic IE1 peptide 170-HFMPT-174, which represents the TCR contact site required and sufficient for sensitization of IE1-specific CD8 T cells [82,83]. Although the L176A mutation is predicted to also reduce the probability of proteasomal C-terminal cleavage [84–86], trace amounts of the mutated peptide may bind to the presenting MHC class-I molecule L^d^, catalyzed by chaperones in the peptide loading complex [87,88]. Such a limited presentation may suffice for sensitization of a fraction of the polyclonal IE1-specific memory CD8 T cells that possess TCRs of particularly high avidity.

### Differential antiviral efficacy of memory CD8 T-cell subsets

Data so far essentially confirmed recent findings reported for AT performed with total memory CD8 T cells [89]. Here we expanded on this study by investigating the differential contributions of memory CD8 T-cell subsets representing activation stages defined by the expression of the cell surface markers CD62L and KLRG1: T central memory cells (TCM) display the cell surface phenotype CD62L^+^KLRG1^-^ and are distinct from naïve T cells by the expression of CD44. T effector-memory cells (TEM) lack expression of CD62L and split into conventional TEM (cTEM) and inflationary TEM (iTEM), distinguished by absence or presence of KLRG1 cell surface expression, respectively (recall S1A Fig).

We were particularly interested in quantitating the antiviral potency of the CD62L^-^KLRG1^+^ iTEM, which are highly activated due to more recent restimulation by the cognate viral antigenic peptide. This is expressed and presented in a stochastic manner during mCMV latency as a result of intermittent transcriptional gene de-silencing events [62]. They expand and thus accumulate over time based on repetitive restimulation, a phenomenon known as MI. Based on their original classification as “short-lived effector cells” (SLEC) [54], iTEM are supposed to interfere with virus replication almost instantly after AT already at the site of infection and thereby prevent virus dissemination to organ sites of CMV disease. In contrast, TCM must first see the cognate epitope for clonal expansion and maturation to effector cells. They therefore might come too late for preventing virus colonization of host tissues and might rather interfere with later intra-tissue virus spread. In fact, this was recently found to be the case for unseparated total memory CD8 T cells [89], but in that earlier study the quantitative subset composition of the viral epitope-specific memory T-cell pool and possible differential contributions of the activation subsets, temporally and spatially, were not addressed. Thus, the previous findings may have reflected the properties of the majority subset, that is, the TCM (recall S1A Fig).

**Fig 3:**
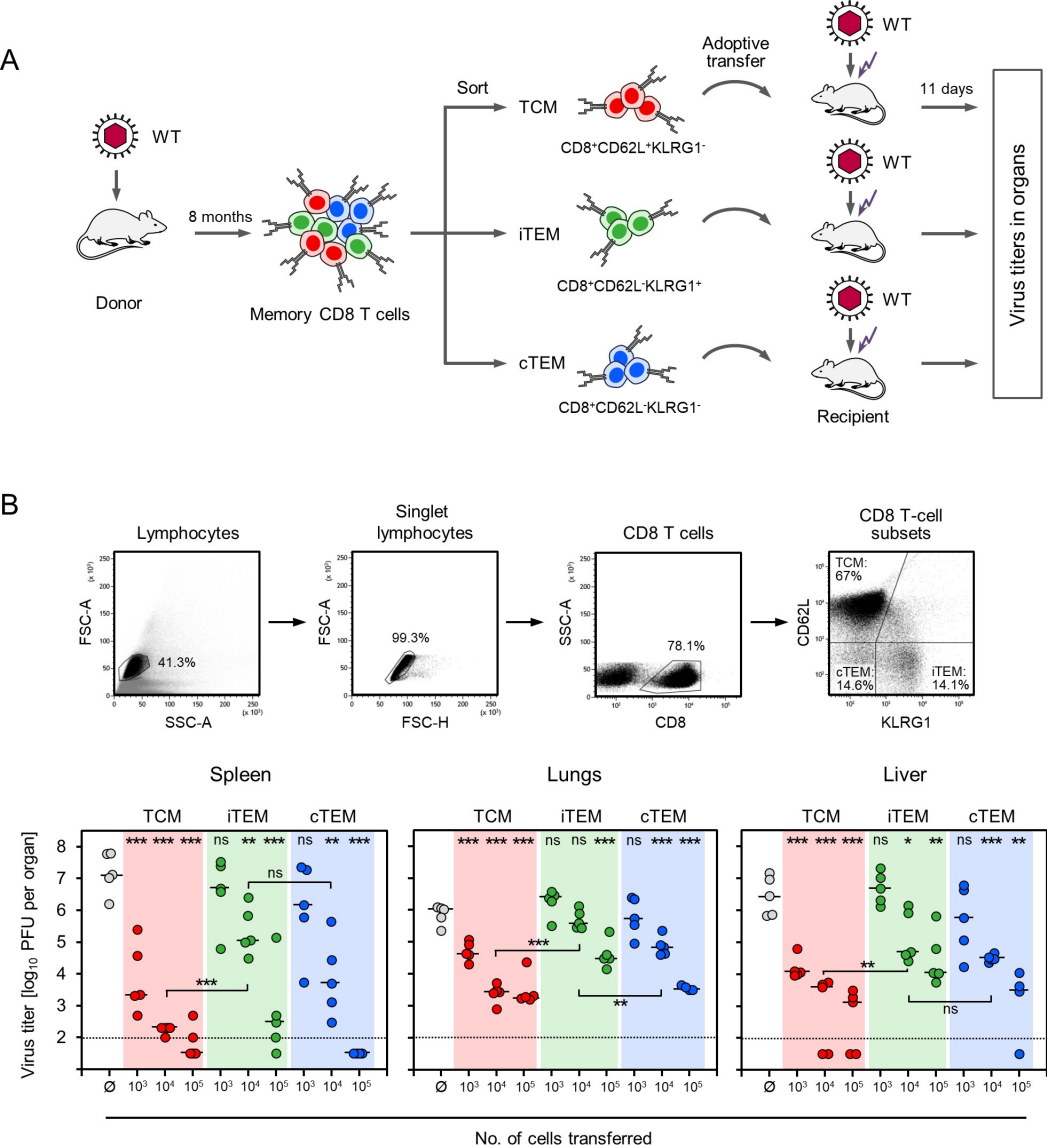
Control of infection by AT of memory CD8 T-cell subsets. (A) Sketch of the experimental protocol. (WT) mCMV-WT. (TCM) T central memory cells. (iTEM) inflationary T effector-memory cells. (cTEM) conventional T effector-memory cells. For AT recipients, see the Legend to Fig 1. (B, upper panel) Gating strategy for the cell sorting. Shown are 2D-dot plots with progressing gates set on the cells indicated. (FSC) forward scatter. (SSC) sideward scatter. (B, lower panel) Control of productive infection in organs of AT recipients by sort-purified donor-derived memory CD8 T-cell subsets. AT was performed with graded donor cell numbers. Ø, no AT performed. Symbols represent individual mice (n = 5 per group) with median values indicated. Data for transferred CD8 T-cell subsets are color-coded as defined in (A). Dotted lines represent the detection limit of the virus plaque assay. (PFU) plaque-forming units. Asterisk-coded statistical significance levels for differences between the AT groups and the no-AT control group (Ø): (*) P< 0.05, (**) P< 0.01, and (***) P< 0.001. (ns) not significant.

For evaluating the contributions of the three subsets separately and normalized to a per-cell basis, they were purified by fluorescence-based cell sorting and tested for their individual antiviral efficacies upon AT into immunocompromised and infected recipient mice (Fig 3A). Two independent experiments (S2 Fig: pilot experiment, and Fig 3B: reproduction in a second experiment with an extended range of cell numbers transferred) were consistent in showing that TCM were the most potent memory cell subset in controlling virus replication in host organs, whereas iTEM were the least potent. Specifically, when compared to the control group of no AT, 1,000 TCM significantly reduced virus replication in all three organs tested, whereas iTEM and cTEM both failed (Fig 3B). With increasing cell numbers, control of infection by iTEM and cTEM was increasingly improved, with a tendency to the favor of cTEM over iTEM. Only upon transfer of 100,000 cells, all three subsets reduced virus replication with statistical significance in all organs tested (Figs 3B and S2B). In conclusion, the ranking in antiviral efficacy was TCM >> cTEM > iTEM.

Survival rates were not determined here, because rescue by AT of virus-specific CD8 T cells is long established [30,78]. It is well known that effective control of viral spread in host tissues prevents lethal histopathology and leads to survival. This is likely to be true irrespective of whether antiviral control is achieved by a low dose of TCM or a high dose of TEM.

### The superior antiviral activity of TCM is not explained by higher numbers of functional viral epitope-specific cells

**Fig 4:**
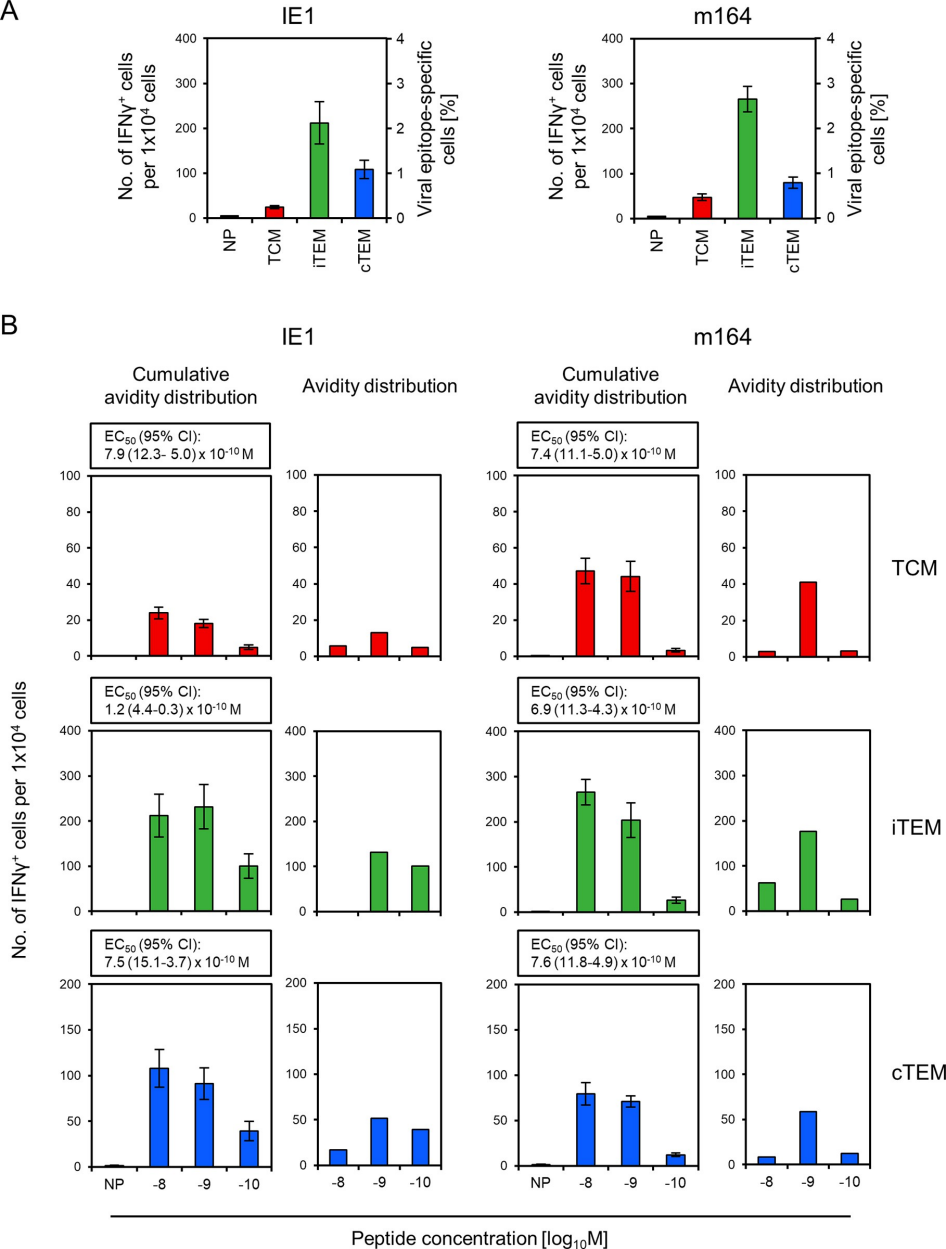
Frequencies and functional avidities of viral epitope-specific memory CD8 T cells differentiated by activation status. (A) Frequencies of viral epitope-specific TCM, iTEM, and cTEM. (Left panel) IE1 peptide-specific cells. (Right panel) m164 peptide-specific cells. Frequencies refer to functional cells responding with secretion of IFNγ in an ELISpot assay to stimulation by P815 cells loaded with the respective antigenic peptide at the saturating concentration of 10^-8^M. (NP) no peptide. (B) Cumulative avidity distributions and deduced Gaussian-like avidity distributions of TCM, iTEM, and cTEM specific for antigenic peptides IE1 and m164, corresponding to (A). Stimulator cells in the ELISpot assay were P815 cells loaded with the respective antigenic peptide in the graded concentrations indicated. (NP) no peptide. (EC_50_ and 95% CI) effective concentration and its 95% confidence interval of antigenic peptide that leads to the half-maximal response of the CD8 T-cell population tested. Throughout, bars represent frequencies determined by intercept-free linear regression analysis. Error bars represent the 95% confidence intervals. Data for transferred CD8 T-cell subsets are color-coded as defined in Fig 3A.

As protection is mediated by epitope-specific CD8 T cells (recall Fig 2), differential antiviral activity of sorted memory CD8 T-cell subsets might simply reflect differences in the specificity composition of the cell pools. We focused our analysis on the frequencies of IE1- and m164-specific cells that account for the majority of viral epitope-specific cells in an unseparated memory cell population (recall Fig 1B). When analyzed for the cell-sorted subsets, frequencies of IE1-and m164-specific IFNγ^+^ functional cells were higher in iTEM (4.8%) and cTEM (1.9%) compared to TCM (0.7%) (Fig 4A). This makes sense, because viral epitope-specific iTEM and cTEM reflect more recent antigen restimulation in response to antigenic mCMV peptides expressed and presented during the latent infection, whereas cells resulting from past encounters with numerous unrelated antigens quantitatively dominate the TCM pool. Notably, this ranking is just opposite to the ranking in antiviral efficacy. Thus, obviously, the failure of 1,000 iTEM or cTEM in controlling infection (Fig 3B) cannot be explained by a shortage of functional epitope-specific cells. Whereas we focused here on functional cells defined by their ability to secrete IFNγ after stimulation with the cognate antigenic peptide, an independent previous experiment revealed 2-4-fold higher frequencies of CD8 T cells carrying IE1- or m164-specific TCRs, compared to functionally defined frequencies, and a subset distribution that varied in the time course after infection (S3 Fig).

These data also lead to a refined view on the protection data (Fig 3) that had revealed a highly significant protection by AT of 1,000 total TCM. As, in this experiment, IE1- and m164-specific cells together accounted for ~0,7% of total TCM, which can be extrapolated to ~1% being specific for all viral epitopes, ~10 transferred viral epitope-specific TCM actually accounted for the observed protection. Note that such numbers show variance and should not be mistaken as being absolutely precise, but they give a decent idea of the scale.

### The poor antiviral activity of iTEM and cTEM is not explained by low TCR avidity

The antiviral efficacy of AT largely depends on the strength and duration of the interaction between peptide-MHC class-I (pMHC-I) complexes presented at the cell surface of infected cells and the cognate TCRs of the CD8 T cells. This determines the intensity of signaling for CD8 T-cell activation and triggering of effector functions. A key parameter is the “structural avidity” measured as the k_off_ rate that quantifies the dissociation of monomeric pMHC-I ligands from the TCRs on living cells [90]. Specifically, the mouse AT model using CTLL selected for high or low “functional avidity” in recognition of the mCMV epitope m164 [50] demonstrated a causal link between high functional avidity, low k_off_ rate, and high protective capacity upon AT [50,90]. Interaction avidity is particularly critical in the case of viruses that express “immune evasion” proteins interfering with the MHC class-I pathway of antigen presentation (for review, see [20]). As we have shown in the mCMV model, these proteins fail to completely prevent but limit antigen presentation and thereby raise the avidity threshold for CD8 T cells to become sensitized (for review, see [91]). So, only high-avidity CD8 T cells recognize infected host tissue cells and protect upon AT when the virus encodes immune evasion proteins. In contrast, cells infected with an immune evasion gene deletion mutant can be recognized also by low-avidity CD8 T cells, resulting in protection upon AT [50,91].

As our study was performed throughout with wild-type (WT) mCMV encoding the full set of known immune evasion proteins operating in the MHC class-I pathway [20], protection rested on high-avidity CD8 T cells. The threshold avidity for protection in the presence of immune evasion proteins can be defined by the capacity of CD8 T cells to recognize cells exogenously loaded with synthetic antigenic peptides at loading concentrations of < 10^−9^ M [50,91]. We therefore first tested if unseparated memory CD8 T cells specific for epitopes IE1 and m164 also fulfill this condition, as it was actually predicted by the already demonstrated protective antiviral function [89]. Indeed, the unseparated memory CD8 T-cell population included cells capable of recognizing target cells loaded with either of the two antigenic peptides at a concentration of 10^−10^ M. The half-maximal effective concentration (EC_50_) values, which describe the population average, were also below the protection threshold value of 10^−9^ M (S4 Fig).

To our knowledge, the question of whether memory CD8 T-cell subsets differ in their functional avidities has never been addressed experimentally. We took into consideration that the poor protection by iTEM and cTEM might result from lower functional avidities compared to TCM. This possible explanation was clearly refuted by the data (Fig 4B). Specifically, as required for the control of infection, all three subsets included cells capable of recognizing the two antigenic peptides at a peptide loading concentration of 10^−10^ M, and also the EC_50_ values were below the protection threshold value of 10^−9^ M for all three subsets and for both immunodominant peptides.

### Only TCM progeny form fully developed NIF, the histological correlate for antiviral protection

From the very low numbers of viral epitope-specific cells required in AT for control of infection in various organs it becomes intuitively clear that protection is not accomplished by an instant effector function of the few initially transferred cells. Instead, protection must result from the effector function of the progeny cell population generated by *in vivo* clonal expansion of the transferred cells [34,89]. As we have shown previously [89], as well as here (Fig 2), AT of unseparated memory cells leads to CD8 T-cell tissue infiltrates that are not randomly distributed but accumulate at infected cells in a micro-anatomical structure, the NIF. By formation of NIF and executing their effector function within the NIF, the infiltrating CD8 T cells confine and eventually resolve tissue infection.

**Fig 5:**
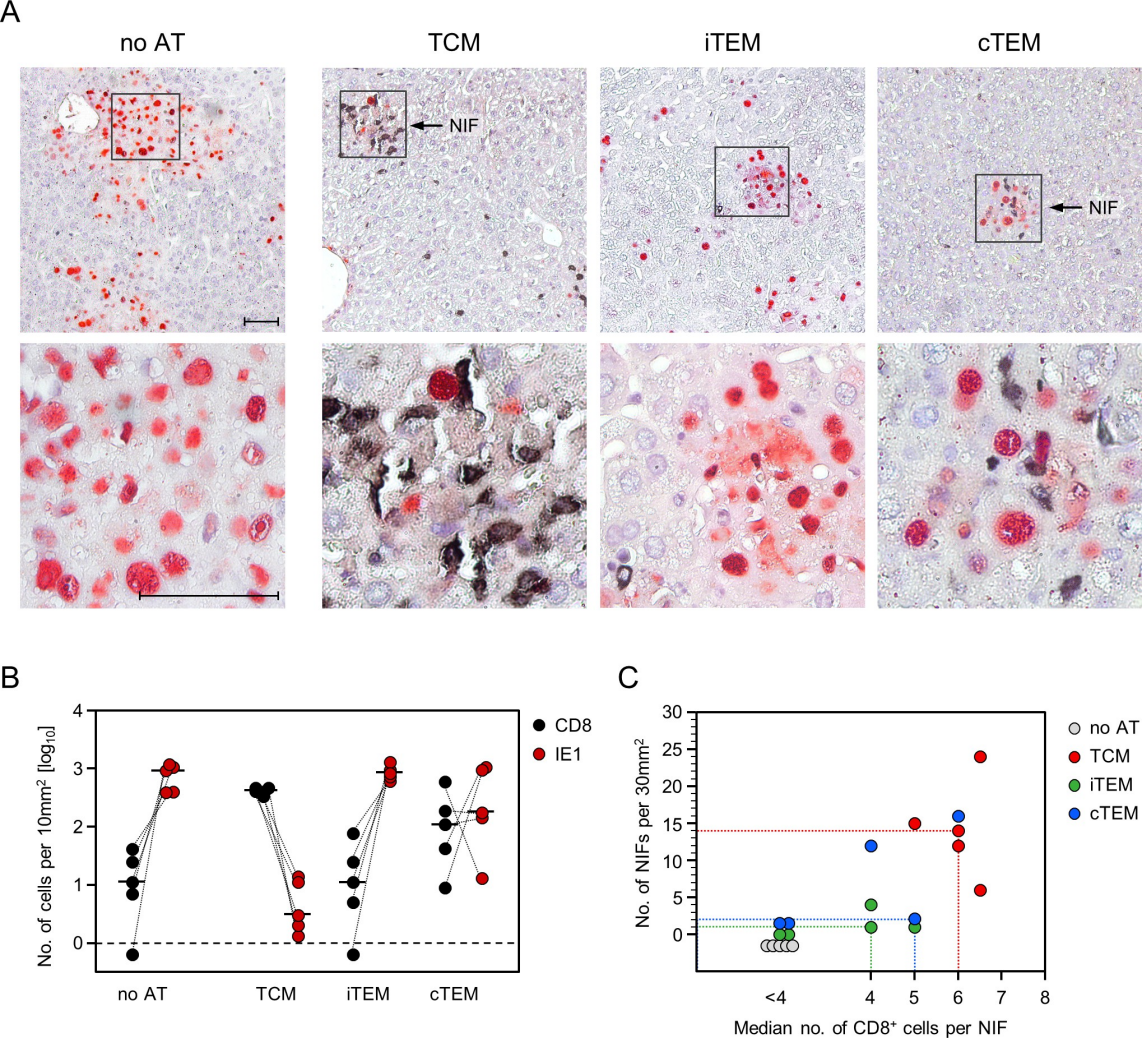
Visualization of liver tissue infection and CD8 T-cell infiltration differentiated by activation status prior to AT. (A) 2C-IHC images of liver tissue sections showing virus control on day 11 after AT by the progeny of 10^3^ TCM, iTEM, or cTEM, corresponding to titers of infectious virus in AT recipients that represent the median values of the experimental groups shown in Fig 3B. Infected liver cells are identified by red staining of the intra-nuclear viral protein IE1. Tissue-infiltrating CD8 T cells are identified by black staining of the CD8a molecule. Light hematoxylin counterstaining reveals the context of liver tissue. (Upper panel images) Low-magnification overviews, showing the confinement of infection by formation of nodular inflammatory foci (NIF) selectively after AT of TCM. Frames demarcate regions of interest resolved to greater detail in the lower panel images. Bar markers represent 50μm and apply to all images. (B) Quantitation of infected IE1^+^ liver cells (red dots) and tissue-infiltrating CD8 T cells (black dots) in representative 10-mm^2^ liver tissue section areas. Symbols represent cell counts for AT recipients (n = 5 per group, corresponding to Fig 3B) tested individually. Linked data are connected by dotted lines, median values are marked. The dashed line indicates the detection limit of the assay. (C) Correlation of the number of NIFs per representative 30-mm^2^ liver tissue section areas (ordinate) with the median number of CD8+ cells per NIF (abscissa), differentiated by transferred CD8 T-cell subset. Symbols represent data from individual mice (n = 5 per group, corresponding to panel B and Fig 3B). The dotted lines mark the AT recipients with the median number of NIFs.

Here we tested the formation of NIF after AT of sorted subsets TCM, iTEM, and cTEM from the experiment shown in Fig 3, all with the same starting cell number of as few as 10^3^ transferred cells (Fig 5). Representative 2C-IHC images of liver tissue sections stained for CD8 and the viral IE1 protein illustrate that fully developed NIF are formed only after AT of TCM, whereas progeny of cTEM form incomplete NIF, and NIF are absent after AT of iTEM (Fig 5A). Quantitation of liver tissue-infiltrating CD8 T cells revealed a rank order of TCM > cTEM > iTEM, anti-correlated to the numbers of infected liver tissue cells in the inverse rank order of TCM < cTEM < iTEM (Fig 5B). A 2-dimensional plot of NIF size, with the number of NIF on the ordinate and the median number of CD8 T cells per NIF on the abscissa, confirmed the ranking of TCM > cTEM > iTEM (Fig 5C). In conclusion, according to all parameters tested, control of infection is most efficient after AT of TCM, as only TCM progeny infiltrate tissue in numbers needed to form fully developed NIF capable of confining the infection.

### The superior protection by AT of TCM is explained by their high *in situ* proliferative potential

The 2C-IHC analysis detecting CD8 T cells and infected cells in their tissue context have revealed the highest numbers of liver tissue-infiltrating CD8 T cells after AT of TCM (Fig 5), although the number of initially transferred cells was the same for all three subsets and even though the number of viral epitope-specific cells was actually lowest in the TCM population (recall Fig 4A). It was therefore reasonable to expect a higher proliferation rate of TCM compared to cTEM and iTEM.

**Fig 6:**
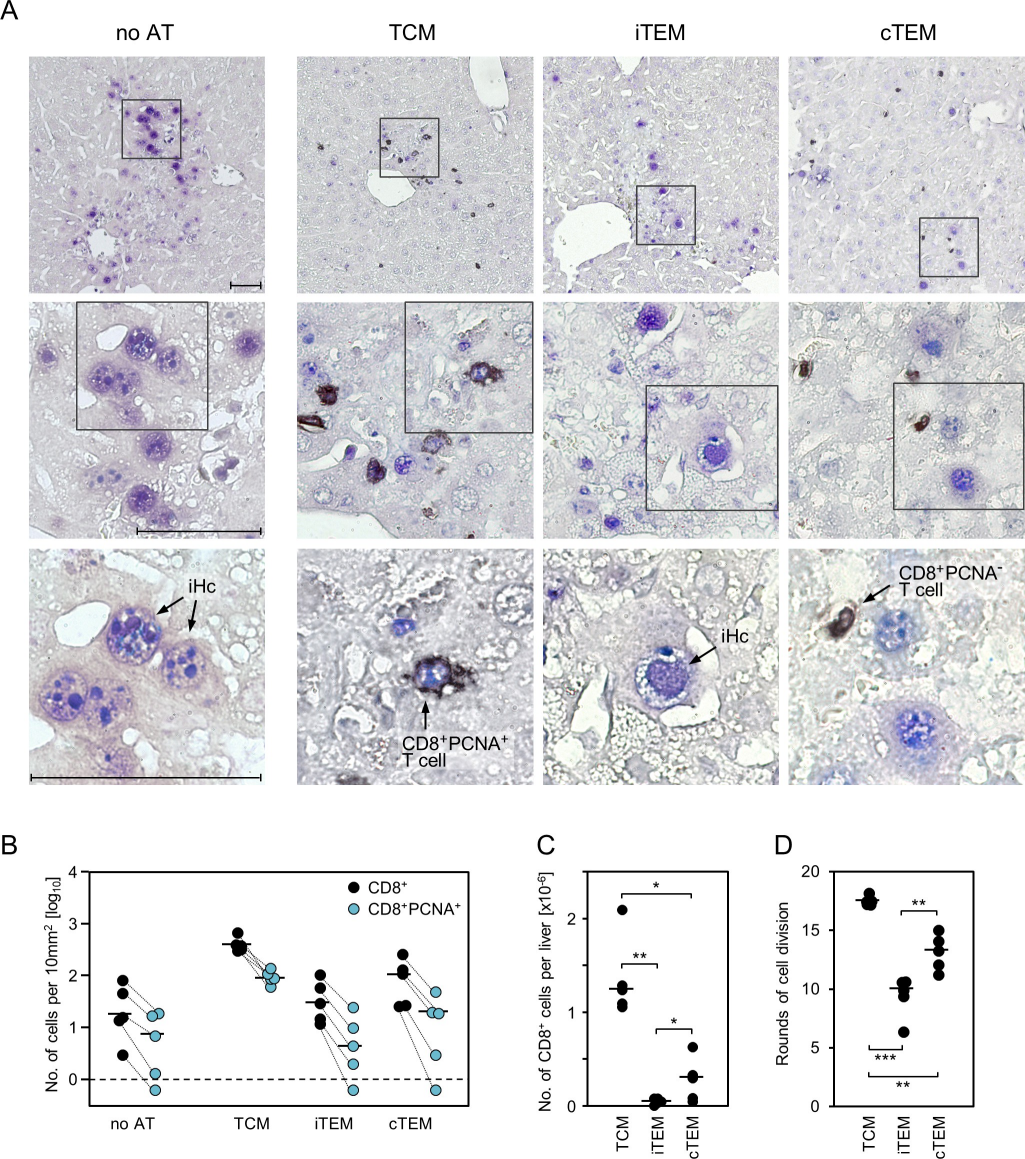
Visualization of extra-lymphoid proliferation and whole-organ quantitation of memory CD8 T-cell progeny differentiated by the activation status prior to AT. (A) 2C-IHC images of liver tissue sections illustrating *in situ* proliferating CD8 T cells on day 11 after AT of 10^3^ TCM, iTEM, or cTEM. (no AT) control group with no AT being performed. Liver tissue-infiltrating CD8 T cells are identified by black staining of the CD8a molecule. The “proliferating cell nuclear antigen (PCNA)” is identified by blue nuclear staining. (CD8^+^PCNA^+^ T cell) Proliferating CD8 T cells are identified by co-expression of intranuclear PCNA, stained in blue, and cytoplasmic as well as membrane CD8a, stained in black. These cells are easy to detect in the TCM group but rarely in the iTEM and cTEM groups. (CD8^+^PCNA^-^ T cell) CD8 T cells not proliferating at the time of analysis. (iHc) productively infected liver cells, most of which are hepatocytes, also express PCNA in their nuclei. Light hematoxylin counterstaining reveals the context of liver tissue. (Upper panel images) Low-magnification overviews. Frames demarcate regions of interest resolved to increasingly greater detail in the center and lower panel images. Arrows point to representative cells of interest. Bar markers represent 50μm throughout. (B) Quantitation of all liver tissue-infiltrating CD8^+^ cells (black dots) and of proliferating CD8^+^PCNA^+^ cells as a subset thereof (blue dots) in representative 10-mm^2^ liver tissue section areas. Symbols represent cell counts for AT recipients (n = 5) tested individually. Linked data are connected by dotted lines, median values are marked. The dashed line indicates the detection limit of the assay. (C) The absolute numbers of CD8 T cells present in the whole liver of the AT recipients on read-out day 11 were calculated by extrapolation based on the numbers counted in the tissue sections. Symbols represent data from mice tested individually (n = 5, corresponding to B), with the median values marked. (D) The numbers of CD8 T-cell divisions that have occurred until the read-out day 11 were calculated based on the numbers of functional, IFNγ-secreting viral epitope-specific cells present in initially transferred 10^3^ cells of the memory CD8 T-cell subsets TCM, iTEM, and cTEM (corresponding to Fig 4A). Symbols represent data for individual AT recipients (n = 5, corresponding to B and C). Median values are marked. Asterisk-coded statistical significance levels for the comparison between the bracketed groups indicated: (*) P< 0.05, (**) P< 0.01, and (***) P< 0.001.

Standard assays for *in vivo* proliferation are based on loss of a fluorescent reporter dye with every cell division [92,93], but this approach requires AT of high cell numbers, and the resolution is limited to few cell divisions until fluorescence intensity falls below the detection limit. To overcome such technical limitations, we used here our previously described approach of a quantitative “input-output” comparison of the number of initially transferred viral epitope-specific CD8 T cells with the absolute number of progeny cells present in a central host organ at defined times after AT [34,89]. CD8 T cells in the stage of cell division were detected *in situ* by 2C-IHC specific for the CD8 molecule, which localizes to the cytoplasm and cell membrane, and the intra-nuclear “proliferating cell nuclear antigen (PCNA)” (Fig 6A, images). PCNA is a credible marker for currently proliferating cells, as it is expressed during the cell cycle in the G1 phase, reaches its maximum in the S phase and declines during the G2/M phase [94–96]. Compared to *ex vivo* detection of proliferation markers, 2C-IHC has the distinct advantage of localizing proliferating cells in infected tissues, thus visualizing them in their micro-anatomical context [97]. To avoid confusion, it must be noted that productively infected cells also express PCNA in the nucleus, reflecting viral DNA replication activity [98]. This poses no problem, as PCNA^+^ infected liver cells do not co-express CD8.

In accordance with the 2C-IHC analysis of CD8 T cells and IE1-expressing infected liver cells shown above (Fig 5), the number of liver-infiltrating CD8 T cells ranked TCM > cTEM > iTEM, and this ranking also applied to proliferating CD8^+^PCNA^+^ cells (Fig 6B). Throughout, CD8^+^PCNA^+^ cells present at the time of analysis represented only a fraction of the tissue-infiltrate CD8 T cells. This is easy to explain, as PCNA^-^ tissue-infiltrate CD8 T cells likely have completed proliferation and were thus back to the cell-cycle G0-phase at the time of analysis. Absolute cell counts, extrapolated from tissue sections to the total liver (for the formula, see [34]), revealed an almost absence of iTEM progeny and a strong predominance of TCM progeny over cTEM progeny (Fig 6C), corresponding to integer median cell division numbers (ranges) of 17 (17–18) for TCM, 10 (6–11) for iTEM, and 13 (11–15) for cTEM (Fig 6D).

## Discussion

In clinical immunotherapy to prevent CMV disease in HCT recipients, providing sufficient cell numbers for AT remains a logistical challenge. Early clinical protocols followed the strategy to expand low numbers of memory CD8 T cells derived from a “CMV-antibody seropositive” donor to high cell numbers in cell culture, that is, by establishing clonal or polyclonal cytolytic T-lymphocyte lines (CTLL) [35,36,52]. Translated from the language of clinicians to the language of viral immunologists, the presence of CMV-specific antibodies is merely an indicator for a past primary CMV infection that has developed into a latent infection, characterized by absence of infectious virus, maintenance of the viral genome in certain cell types, and presence of CMV-specific immunological memory (reviewed in [99], and recently updated for mCMV in [64,65]).

In the early days, clinical AT focused entirely on CTLL specific for the abundant hCMV tegument protein pp65/UL83 [35,36], because the use of virions for restimulation in cell culture had led to the selection to the favor of pp65/UL83-specific CTLL [100–103]. Unbiased determination of the specificity spectrum of the human memory CD8 T-cell response to hCMV by viral genome-wide antigenicity screening confirmed pp65/UL83 as a significant source of antigenic peptides but, when based on broad HLA coverage, revealed an antigenicity of most hCMV ORFs and still of several ORFs for the HLA set of any individual [104]. Accordingly, for each HLA class-I matched donor-recipient pair, CD8 T-cells of several specificities qualify as candidates for use in AT.

An important contribution of the mouse model was the finding that expansion of memory CD8 T cells to high numbers of effector cells in cell culture comes at the cost of a loss of protective activity per cell, thus negating the effort to establish CTLL [48,49]. Clinical data also revealed a superior protection capacity of *ex vivo* isolated memory CD8 T cells [38,39,51] when compared to published experience with CTLL, but only the mouse model allowed the “Proof of Concept” by a direct comparison between *ex vivo* sort-purified memory CD8 T cells and CTLL specific for the same viral epitope and tested in parallel in the same experiment. This comparison revealed more than two orders of magnitude higher protective capacity of the memory cells [48,49]. Our data presented here suggest that loss of proliferative capacity by terminal differentiation to effector cells explains the low per-cell antiviral efficacy of CTLL compared to memory CD8 T cells capable of expanding within the AT recipient.

While the use of memory CD8 T cells for clinical AT is meanwhile standard, less attention is paid to potentially different properties of memory cell activation and differentiation subsets, specifically of central memory cells (TCM) and effector-memory cells (TEM), which can be subdivided into conventional TEM (cTEM) and inflationary TEM (iTEM). “Stemness” with high self-renewal capacity associated with a high proliferation rate is a reported advantage of CD62L^+^ TCM compared to CD62L^-^ TEM in mouse models of bacterial pathogen-specific AT [39,105], and, in analogy, low-dose clinical AT of hCMV-specific human CD8^+^ T cells into HCT patients led to a vigorous expansion of the transferred cells [39]. The clinical hCMV part of the study by Stemberger and colleagues [39], however, depended on compassionate-use settings that did not allow a distinction between TCM and TEM. Nonetheless, based on these studies, one can take it for granted that TCM generally have a superior proliferation capacity. On the other hand, a protective effect of TCM comes with delay, whereas, as the name “effector-memory cell” indicates, TEM might have the advantage of a more rapid antiviral effect.

In latent CMV infections, the viral genomes are not completely silenced. Instead, episodes of viral gene desilencing lead to limited and transient transcription that does not follow the coordinated gene expression cascade of the productive viral cycle [106–109]. Linking this to immune surveillance of latent infection, the murine model has shown stochastic and transient expression also of viral genes that code for antigenic peptides [61,62]. Their presentation on the surface of latently infected cells, meanwhile identified as endothelial cell types, is proposed to drive memory inflation (MI) [56,62]. The stochastic nature of the transcription episodes is perfectly reflected by stochastic clonal expansions of virus epitope-specific CD8 T cells [110] and explains the variance in viral epitope-specific CD8 T-cell reconstitution dynamics between different HCTs performed with an identical protocol [25].

MI is defined by expansion of the pool of KLRG1^+^CD62L^-^ TEM, previously referred to as “short-lived effector cells (SLEC)” [54], which we have proposed to re-name inflationary TEM (iTEM) in distinction from non-inflationary KLRG1^-^CD62L^-^ conventional TEM (cTEM) [53]. MI is favored by conditions of primary infection that lead to a high load of latent viral DNA, since a high number of latent viral genomes as templates obviously increases the probability for episodes of antigen-coding transcription. One parameter, experimentally shown to favor MI in the immunocompetent mouse model, is the initial dose of infection [111]. However, regardless of the initial dose of infection, which is probably always very low in humans, subsequent virus spread in the host that largely depends on the immune status at the time of infection and which is high in the immunologically immature or immunocompromised host, is likely even more important for establishing a high latent viral genome load favoring MI [72,112]. Dependence on the individual infection history, defining the magnitude of productive infection and thus also the latent viral DNA load, explains why MI is not consistently observed in humans ([113], for a commentary, see [114]).

The human counterpart of iTEM, that is, the cells expanding during MI and thus defining MI, show an “advanced differentiation phenotype” that also includes high expression of KLRG1 and low expression of CD62L [115]. In both, human and murine latent CMV infections, the inflationary CD8 T cells are functional (reviewed in [116]). Specifically, in mCMV, MI during viral latency was originally described as expansion of CD62L^-^ cells capable of secreting IFNγ upon stimulation by antigenic peptide presentation [117], and SLEC/iTEM were found not to be terminally differentiated effector cells but to proliferate upon AT [54]. Based on their high numbers under conditions of MI, facilitating their isolation, and on their functionality and proliferative potential, iTEM were promising candidates for controlling CMV infection. As far as we are aware of, although iTEM were proposed to surveil viral latency by sensing and terminating productive viral reactivation [61], their capacity to control acute infection was never tested in an AT model.

Controlled studies comparing the protective efficacies of memory CD8 T-cell subsets in parallel by AT into recipient hosts are not feasible in humans, because individual HCT patients differ in their genetics and progress of hematopoietic reconstitution, latent virus strain(s)/clinical isolates that can differ in host-cell tropism [1,67,68,118–121], latent viral genome load depending on the individual history of infection, time of virus reactivation onset and organ site of reactivation, as well as in the extent of inter- as well as intra-tissue virus spread. In addition, there is no way to find identical cohorts of donors for an unbiased comparison of different cell populations, because all individuals differ in their memory-shaping infection history, including latent virus strain and epitope-specificity composition of the memory cell pool. Every single one of these unavoidable variables in both AT donor and recipient renders a quantitative comparison between memory CD8 T-cell subsets by clinical AT a lottery game. It is this type of question that can reliably be answered only in a reductionist animal model [21].

And the answer was clear: under conditions of low-dose AT, performed in absence of HCT to reduce the number of variables, only TCM were able to clear productive infection by infiltrating infected host tissues in numbers high enough to form NIF, the micro-anatomical correlate of protection. All three memory CD8 T-cell subsets were able to control infection after high-dose AT. Protection by cTEM, and in particular by iTEM at high doses may indeed reflect their more rapidly exerted antiviral effector function, but this is of little help for clinical AT where the provision of high cell numbers is the logistically limiting factor for AT. Notably, the number of iTEM required for control of the infection is strikingly similar to the experience made previously with CTLL [48,49]. In fact, iTEM and the majority of the cells of a CTLL share the “advanced differentiation phenotype” of high KLRG1 and low CD62L expression. Notably, as shown previously for a short-term CD8^+^ CTLL specific for the IE1 peptide and propagated by repetitive restimulation in cell culture, the CTLL population split into a majority of iTEM-like KLRG1^+^CD62L^-^ cells and a minority of cTEM-like KLRG1^-^CD62L^-^cells [50]. These are precisely the two phenotypes that are of low efficacy in AT, with iTEM < cTEM (this report). We thus would like to put forward the interpretation that CD8 T cells repetitively restimulated by stochastically expressed and presented antigenic peptides in the latently infected host in a sense resemble CTLL, with the difference that CTLL also contain terminally-differentiated, cytolytic TEC, whereas *ex vivo* isolated viral epitope-specific CD8 T cells are cytolytically active only in the acute phase of infection but no longer during latent infection [122].

Attempting to define the reason for the higher protective activity of TCM in low-dose AT compared to iTEM and cTEM, we have excluded a higher frequency of viral epitope-specific cells or a higher functional avidity in recognizing antigenic peptides presented on infected cells. The most distinctive difference between TCM and both subsets of TEM was the superior viral epitope-specific infiltration into infected host tissues and the formation of NIF, as shown here exemplarily for the liver. This cannot be explained by a more efficient homing of the initially transferred CD62L^+^ TCM to non-lymphoid tissues, because CD62L, also known as L-selectin, is a homing receptor expressed by recirculating cells for mediating their temporal homing to lymphoid tissues. It appears that TCM must first be activated by antigen encounter to convert to CD62L^-^ TEM for infiltrating non-lymphoid tissues [123,124]. In accordance with this, as we have shown recently for the location of viral epitope-specific CD8 T cells within latently infected lungs [125], CD62L^-^ TEM efficiently migrate from the intravascular compartment (IVC) to the extravascular compartment (EVC), the lung parenchyma, where in particular cTEM were found enriched. In contrast, CD62L^+^ TCM are absent in the EVC, which is best explained by loss of CD62L and conversion to TEM during transmigration of the lung endothelium. From all this we conclude that TEM exogenously administered to the IVC by AT will also efficiently infiltrate infected host tissues.

So, what remains as an explanation for the much better antiviral control by AT of TCM is their superior proliferation capacity that leads to a massive expansion of the pool of protective TEC. It is a strength of our approach that we visualized intra-tissue virus spread and its prevention by infiltrating, NIF-forming CD8 T cells in 2C-IHC. Notably, using PCNA as a credible marker for proliferation, we could demonstrate an *in situ* proliferation of CD8 T cells at a non-lymphoid site of viral pathogenesis.

An "input-output" quantification of the progeny of the transferred cells allowed us to provide an estimate of the number of cell cycles undergone after AT (see Fig 6D). This estimate is an approximation because, for simplicity, the original cell count for AT included only functional IFNγ-producing cells specific for the two immunodominant epitopes, and because quantification of progeny was based only on evaluation of the liver. If the calculation were based on the number of cells expressing viral epitope-specific TCRs (S3 Fig), the estimate would be lower by 1 to 2 proliferation cycles. On the other hand, since progeny of the transferred cells distribute not only to the liver, the estimate must be revised upward. The two corrections therefore go in opposite directions, so that their omission does not affect the relevant message of ranking the proliferation capacity in the order of TCM >> cTEM > iTEM.

Although the absolute number of iTEM progeny in the liver was almost negligible, our data are not conflicting with the finding by Snyder and colleagues [54], who have shown that fluorescence-labeled iTEM proliferate upon AT. We have estimated 10 (6–11) cell cycles for iTEM and 17 (17–18) cell cycles for TCM. This does not sound like a dramatic difference, unless one considers the mathematical nature of exponential functions: high absolute numbers are generated by the late cell divisions, whereas the early cell divisions make only a small contribution.

In conclusion, low-dose AT of iTEM or cTEM in HCT patients is no promising option, as protection against CMV disease depends on vigorous expansion of virus-specific TCM. For clinical protocols, our data should not be mistaken to suggest the use of a small number of TCM for AT, because many conditions in HCT recipients may interfere with the efficient expansion of any memory CD8 T-cell subset. As we have shown recently, therapeutic vaccination of recipients can improve antiviral protection by promoting the post-AT expansion of low numbers of transferred CD8 T cells [34]. We also do not recommend incurring the logistical expense of purifying TCM for AT, but our findings definitely refute the idea of performing AT with low numbers of purified TEM.

## Materials and methods

### Ethics statement

Animal experiments were performed in accordance with the national animal protection law (Tierschutzgesetz (TierSchG)), animal experiment regulations (Tierschutz-Versuchstierverordnung (TierSchVersV)), and the recommendations of the Federation of European Laboratory Animal Science Association (FELASA). The experiments were approved by the ethics committee of the Landesuntersuchungsamt Rheinland-Pfalz, permission numbers 177-07/G14-1-015 and 177-07/G19-1-049.

### Mice, viruses, and route of infection

Female BALB/cJ (haplotype H-2^d^) mice were bred and housed under specified-pathogen-free (SPF) conditions by the Translational Animal Research Center (TARC) at the University Medical Center of the Johannes Gutenberg-University Mainz. Immunocompetent CD8 T-cell donors and immunocompromised recipients were used at an age of 8-to-12 weeks.

mCMV (strain Smith, ATCC VR-1399) was used as wild-type virus (mCMV-WT). BAC-derived recombinant viruses mCMV-IE1-L176A and the corresponding revertant mCMV-IE1-A176L were described previously [61]. All viruses were propagated in cell culture and purified by standard methods [126]. Intraplantar infection was performed by injection of 10^5^ plaque-forming units (PFU) of the respective virus into the left hind footpad.

### Immunomagnetic enrichment of memory CD8 T cells and fluorescence-based cell sorting

Immunocompetent BALB/cJ mice were infected with mCMV-WT for use as T-cell donors. After 8 months, CD8 T cells derived from a pool of 10–12 spleens were enriched either by immunomagnetic positive selection [127], or, in cases of subsequent fluorescence-based cell sorting, by immunomagnetic negative selection using the MagniSort mouse CD8 T-cell enrichment kit (catalog no. 8804-6822-74; eBioscience), following the manufacturer’s instructions.

Memory CD8 T-cell subsets were isolated by fluorescence-based cell sorting after staining of CD8 and activation markers CD62L and KLRG1, using FITC-conjugated anti-CD8a (clone 53–6.7; eBioscience), PE/Dazzle594-conjugated anti-KLRG1 (clone 2F1; BioLegend), and PE-Cy7-conjugated anti-CD62L (clone MEL-14; eBioscience). Sort gates were set on CD8 T cells and CD8 T-cell subsets TCM (CD62L^+^KLRG1^-^), iTEM (CD62L^-^KLRG1^+^), and cTEM (CD62L^-^KLRG1^-^). IE1-specific memory CD8 T cells were purified by fluorescence-based cell sorting using PE-conjugated, IE1 peptide-folded MHC-I dextramers H-2Ld/YPHFMPTNL (Immudex, Copenhagen, Denmark). Analyses and cell sorting were performed with flow cytometer BD FACSAria I and FACSDiva analysis software (BD Biosciences).

### Cytofluorometric (CFM) analyses

Enrichment of CD8 T cells by immunomagnetic selection was documented by CFM quantitation of TCRβ^+^CD8^+^CD4^-^ lymphocytes. Unspecific staining was blocked with unconjugated anti-FcγRII/III antibody (anti-CD16/CD32, clone 93; BioLegend). Specific staining for 3-color CFM analysis was performed with PE-conjugated anti-TCRβ (clone H57-597; BD Bioscience), PE-Cy5-conjugated anti-CD8a (clone 53–6.7; eBioscience), and FITC-conjugated anti-CD4 (clone GK1.5; BioLegend) antibodies. For distinguishing antigen-experienced memory CD8 T-cell subsets from naïve CD8 T cells, a 4-color CFM analysis was performed with PE-Cy7-conjugated anti-CD44 (clone IM7; eBioscience), PE/Dazzle594-conjugated anti-CD8a (clone 53–6.7; BioLegend), FITC-conjugated anti-KLRG1 (clone 2F1; eBioscience), and PE-conjugated anti-CD62L (clone MEL-14; eBioscience) antibodies. All analyses were performed with flow cytometer Cytomics FC500 and CXP analysis software (Beckman Coulter).

### Adoptive transfer (AT) of memory CD8 T cells

For AT of donor-derived memory CD8 T cells or subsets thereof, 8-week-old BALB/cJ recipients were immunocompromised by hemato-ablative total-body γ-irradiation with a single dose of 6.5 Gy, followed 4 hours later by intraplantar infection with mCMV-WT. AT was performed as a pre-emptive immunotherapy by intravenous cell infusion 2 hours after infection. In the specific case of AT of IE1 epitope-specific memory CD8 T cells, recipients were infected either with mutant virus mCMV-IE1-L176A or revertant virus mCMV-IE1-A176L. Throughout, mice left without AT served as controls for unrestricted viral replication.

### Peptides and quantitation of functional epitope-specific memory CD8 T cells

Viral epitopes corresponding to antigenic peptides presented by MHC class-I molecules K^d^, D^d^, and L^d^ are derived from the mCMV ORFs m18, M83, M84, M105, m123/IE1, m145, and m164 (amino acid sequences and presenting MHC class-I molecules listed in [50]). Custom peptide synthesis with a purity of > 80% was performed by JPT Peptide Technologies.

Immunomagnetically-purified CD8 T cells and sorted subsets thereof, derived from pooled spleens of AT donor mice latently infected with mCMV-WT, served as responder cells in IFNγ-based enzyme-linked immunospot (ELISpot) assays ([128], and references therein). In essence, for quantitating functional, mCMV epitope-specific memory CD8 T cells, the corresponding synthetic peptides were exogenously loaded on P815 (H-2^d^) mastocytoma cells at the molar concentrations indicated, for serving as stimulator cells in the assay. Graded numbers of responder cells were seeded with the peptide-loaded stimulator cells in triplicate microcultures. After 18 hrs of incubation, spots were counted using the ImmunoSpot S4 Pro Analyzer (Cellular Technology Limited).

### ELISpot assay calculations and determination of avidity distributions

Frequencies (most probable numbers, MPN) of cells responding in the ELISpot assay and the corresponding 95% confidence intervals were calculated by intercept-free linear regression analysis from the linear portions of regression lines based on spot counts from triplicate assay cultures for each of the graded cell numbers seeded [128]. Calculations were performed with SPSS Statistics, Version 23.

Cumulative avidity plots show the measured frequencies of cells responding at the indicated peptide concentration and all lower concentrations. Based on MPN and the corresponding upper and lower 95% confidence limits, half-maximal effective concentration (EC_50_) values, representing peptide concentrations that result in the half-maximal response of the cell population, were calculated with Quest Graph EC_50_ Calculator (AAT Bioquest, Inc.; retrieved from https://www.aatbio.com/tools/ec50-calculator). Gaussian-like avidity distributions reveal frequencies of cells with an avidity defined precisely by the peptide concentration indicated. These are deduced from the cumulative avidity distribution values by plotting the response increments between a certain peptide concentration and the next lower peptide concentration [129].

### Visualization of tissue infection, CD8 T-cell infiltration, and CD8 T-cell proliferation

At 11 days after AT, infectious virus in spleen, lungs, and liver was quantitated in whole organ homogenates by a virus plaque assay performed on monolayers of mouse embryo fibroblasts under conditions of “centrifugal enhancement of infectivity” ([127], and references therein).

To visualize and quantitate infection and CD8 T-cell infiltration in the micro-anatomical context of liver tissue, infected cells and CD8 T cells were identified in tissue sections by 2C-IHC staining of the intra-nuclear viral immediate-early (IE) protein IE1 in red color and the CD8a molecule in black [89]. To detect *in situ* proliferating CD8 T cells, 2C-IHC was used for visualizing cells co-expressing the CD8a molecule (black staining, see above) and PCNA (blue staining). Blue staining of PCNA was achieved by using a species-cross reactive mouse IgG2a-kappa monoclonal antibody directed against PCNA (clone PC10; BD Bioscience) and the Vector Blue Substrate Kit, Alkaline Phosphatase (AP) (catalog no. SK-5300; Vector Laboratories).

### Quantitation of infected tissue cells and tissue-infiltrating CD8 T cells

Total numbers of infected cells and of tissue-infiltrating CD8 T cells were calculated from cells counted in representative tissue sections and extrapolated to the whole organ by using a mathematical formula that corrects for overestimation when the diameter of the counted object is > the thickness of the tissue section (for a detailed explanation, see [34]).

### Calculation of the number of cell divisions

The number of cell divisions was calculated as *n* = log_2_ [*N(t)/N(0)*], where *N(t)* = total number of tissue-infiltrate CD8 T cells per whole organ at time *t* after AT and *N(0)* = number of initially (*t* = 0) transferred viral epitope-specific cells of CD8 T-cell subsets TCM, iTEM, and cTEM.

### Statistical analysis

To evaluate statistical significance of differences between two independent sets of data, the two-sided unpaired t-test with Welch’s correction of unequal variances was used. Differences were considered statistically significant for P-values (*) <0.05, (**) <0.01, and (***) <0.001. Calculations were performed with Graph Pad Prism 10.1 (Graph Pad Software, San Diego, CA).

## Supporting information

S1 Fig Cell surface phenotypes of CD8 T cells. (A) CFM analyses of cell surface marker expression by CD8 T cells derived from the spleen of age-matched uninfected BALB/c mice (upper panels) and of memory CD8 T cells derived from the spleen of BALB/c AT donor mice in the stage of latent infection at 8 months after priming by infection with mCMV-WT (lower panels). Shown are color-coded 2D fluorescence density plots for the cell surface marker combinations indicated, with red and blue color representing highest and lowest cell numbers, respectively. Gates were set on CD8 T cells in the SSC (sideward scatter) versus CD8 plots. (TCM) T central memory cells. (cTEM) conventional T effector-memory cells. (iTEM) inflationary T effector-memory cells. (B) CFM analyses documenting the successful enrichment of CD8 T cells by immunomagnetic cell sorting.
(TIF)

S2 Fig Control of infection by AT of total memory CD8 T cells and activation subsets: pilot experiment. (A) AT of unseparated memory CD8 T cells. For details, see the Legend of Fig 1C in the body of the text. (B) AT of memory CD8 T-cell subsets. (TCM) T central memory cells. (cTEM) conventional T effector-memory cells. (iTEM) inflationary T effector-memory cells. For details, see the Legend of Fig 3B in the body of the text. Asterisk-coded statistical significance levels for differences between the AT groups and the no-AT control group (Ø): (**) P< 0.01, and (***) P< 0.001. (ns) not significant.
(TIF)

S3 Fig Comparison of frequencies of viral epitope-specific IFNγ-secreting and cognate TCR-expressing memory CD8 T cells. Data result from a reanalysis of a previously published experiment [53] with a different focus of interpretation. BALB/c AT donor mice were infected with mCMV-WT, and immunomagnetically enriched spleen-derived memory CD8 T cells were tested at the times indicated. (Left column) Frequencies of functional IE1 and m164 epitope-specific cells among total memory CD8 T cells determined by the ELISpot assay. (Right colums) Frequencies of memory CD8 T cells, total or differentiated by activation subset, expressing IE1 and m164 epitope-specific TCRs detected by CFM analysis (for the method, see [53]). (TCM) T central memory cells. (cTEM) conventional T effector-memory cells. (iTEM) inflationary T effector-memory cells. Error bars are indicated.
(TIF)

S4 Fig Frequencies and functional avidities of viral epitope specific memory CD8 T cells. Shown are cumulative avidity distributions and deduced Gaussian-like avidity distributions of unseparated memory CD8 T cells specific for antigenic peptides IE1 and m164. For details, see the Legend of Fig 4 in the body of the text.
(TIF)

## References

[1] Davison AJ, Holton M, Aidan D, Dargan DJ, Gatherer D, Hayward GS. Comparative genomics of primate cytomegaloviruses. In: Reddehase MJ, editor. Cytomegaloviruses: from molecular pathogenesis to intervention. Norfolk: Caister academic press; 2013. pp. 1–22.

[2] Ho M. The history of cytomegalovirus and its diseases. Med Microbiol Immunol. 2008;197: 65–73. doi: 10.1007/s00430-007-0066-x 18087722

[3] Boppana SB, Britt WJ. Synopsis of clinical aspects of human cytomegalovirus disease. In: Reddehase MJ, editor. Cytomegaloviruses: from molecular pathogenesis to intervention. Norfolk: Caister academic press; 2013. pp. 1–25.

[4] Griffiths P, Reeves M. Pathogenesis of human cytomegalovirus in the immunocompromised host. Nat Rev Microbiol. 2021;19: 759–773. doi: 10.1038/s41579-021-00582-z 34168328 PMC8223196

[5] Adler SP, Nigro G. Clinical cytomegalovirus research: congenital infection. Cytomegaloviruses: from molecular pathogenesis to intervention. Norfolk: Caister academic press; 2013. pp. 55–72.

[6] Cannon MJ, Grosse SD, Fowler KB. The epidemiology and public health impact of congenital cytomegalovirus infection. Cytomegaloviruses: from molecular pathogenesis to intervention. Norfolk: Caister academic press; 2013. pp. 26–48.

[7] Seo S, Boeckh M. Clinical cytomegalovirus research: haematopoietic cell transplantation. Cytomegaloviruses: from molecular pathogenesis to intervention. Norfolk: Caister academic press; 2013. pp. 335–379.

[8] Emery VC. Relative importance of cytomegalovirus load as a risk factor for cytomegalovirus disease in the immunocompromised host. In: Scholz M, Rabenau HF, Doerr HW, Cinatl J, editors. Monographs in Virology. Basel: Karger; 1997. pp. 288–301. doi: 10.1159/000061707

[9] Emery VC, Milne RSB, Griffiths PD. Clinical cytomegalovirus research: liver and kidney transplantation. Cytomegaloviruses: from molecular pathogenesis to intervention. Norfolk: Caister academic press; 2013. pp. 229–309.

[10] Krowka MJ, Rosenow EC, Hoagland HC. Pulmonary complications of bone marrow transplantation. Chest. 1985;87: 237–246. doi: 10.1378/chest.87.2.237 2981658

[11] Klotman ME, Hamilton JD. Cytomegalovirus pneumonia. Semin Respir Infect. 1987;2: 95–103. 2827281

[12] Quabeck K. The lung as a critical organ in marrow transplantation. Bone Marrow Transplant. 1994;14 Suppl 4: S19–28. 7728120

[13] Riddell SR. Pathogenesis of cytomegalovirus pneumonia in immunocompromised hosts. Semin Respir Infect. 1995;10: 199–208. 8668847

[14] Smith MG. Propagation in tissue cultures of a cytopathogenic virus from human salivary gland virus (SGV) disease. Proc Soc Exp Biol Med. 1956;92: 424–430. doi: 10.3181/00379727-92-22498 13350368

[15] Azab W, Dayaram A, Greenwood AD, Osterrieder N. How host specific are herpesviruses? Lessons from herpesviruses infecting wild and endangered mammals. Annu Rev Virol. 2018;5: 53–68. doi: 10.1146/annurev-virology-092917-043227 30052491

[16] Ostermann E, Loroch S, Qian Z, Sickmann A, Wiebusch L, Brune W. Activation of E2F-dependent transcription by the mouse cytomegalovirus M117 protein affects the viral host range. PLoS Pathog. 2018;14: e1007481. doi: 10.1371/journal.ppat.1007481 30532172 PMC6301716

[17] Benedict CA, Crozat K, Degli-Esposti MA, Dalod M. Host genetic models in cytomegalovirus immunology. In: Reddehase MJ, editor. Cytomegaloviruses: from molecular pathogenesis to intervention. Norfolk: Caister academic press; 2013. pp. 258–284.

[18] Redwood AJ, Shellam GR, Smith LM. Molecular evolution of murine cytomegalovirus genomes. In: Reddehase MJ, editor. Cytomegaloviruses: from molecular pathogenesis to intervention. Norfolk: Caister academic press; 2013. pp. 23–37.

[19] Mozzi A, Biolatti M, Cagliani R, Forni D, Dell’Oste V, Pontremoli C, et al. Past and ongoing adaptation of human cytomegalovirus to its host. PLoS Pathog. 2020;16: e1008476. doi: 10.1371/journal.ppat.1008476 32384127 PMC7239485

[20] Becker S, Fink A, Podlech J, Reddehase MJ, Lemmermann NA. Host-adapted gene families involved in murine cytomegalovirus immune evasion. Viruses. 2022;14: 128. doi: 10.3390/v14010128 35062332 PMC8781790

[21] Reddehase MJ, Lemmermann NAW. Mouse model of cytomegalovirus disease and immunotherapy in the immunocompromised host: predictions for medical translation that survived the “Test of Time.” Viruses. 2018;10: 693. doi: 10.3390/v10120693 30563202 PMC6315540

[22] Sissons JGP, Wills MR. How understanding immunology contributes to managing CMV disease in immunosuppressed patients: now and in future. Med Microbiol Immunol. 2015;204: 307–316. doi: 10.1007/s00430-015-0415-0 25896527

[23] Podlech J, Holtappels R, Wirtz N, Steffens HP, Reddehase MJ. Reconstitution of CD8 T cells is essential for the prevention of multiple-organ cytomegalovirus histopathology after bone marrow transplantation. J Gen Virol. 1998;79: 2099–2104. doi: 10.1099/0022-1317-79-9-2099 9747717

[24] Podlech J, Holtappels R, Pahl-Seibert MF, Steffens HP, Reddehase MJ. Murine model of interstitial cytomegalovirus pneumonia in syngeneic bone marrow transplantation: persistence of protective pulmonary CD8-T-cell infiltrates after clearance of acute infection. J Virol. 2000;74: 7496–7507. doi: 10.1128/jvi.74.16.7496-7507.2000 10906203 PMC112270

[25] Holtappels R, Lemmermann NAW, Podlech J, Ebert S, Reddehase MJ. Reconstitution of CD8 T cells protective against cytomegalovirus in a mouse model of hematopoietic cell transplantation: dynamics and inessentiality of epitope immunodominance. Front Immunol. 2016;7: 232. doi: 10.3389/fimmu.2016.00232 27379095 PMC4905951

[26] Holtappels R, Schader SI, Oettel O, Podlech J, Seckert CK, Reddehase MJ, et al. Insufficient antigen presentation due to viral immune evasion explains lethal cytomegalovirus organ disease after allogeneic hematopoietic cell transplantation. Front Cell Infect Microbiol. 2020;10: 157. doi: 10.3389/fcimb.2020.00157 32351904 PMC7174590

[27] Gezinir E, Podlech J, Gergely KM, Becker S, Reddehase MJ, Lemmermann NAW. Enhancement of antigen presentation by deletion of viral immune evasion genes prevents lethal cytomegalovirus disease in minor histocompatibility antigen-mismatched hematopoietic cell transplantation. Front Cell Infect Microbiol. 2020;10: 279. doi: 10.3389/fcimb.2020.00279 32582572 PMC7296086

[28] Reddehase MJ. Mutual Interference between cytomegalovirus and reconstitution of protective immunity after hematopoietic cell transplantation. Front Immunol. 2016;7: 294. doi: 10.3389/fimmu.2016.00294 27540380 PMC4972816

[29] Reddehase MJ, Holtappels R, Lemmermann NAW. Consequence of histoincompatibility beyond GvH-reaction in cytomegalovirus disease associated with allogeneic hematopoietic cell transplantation: change of paradigm. Viruses. 2021;13: 1530. doi: 10.3390/v13081530 34452395 PMC8402734

[30] Reddehase MJ, Weiland F, Münch K, Jonjic S, Lüske A, Koszinowski UH. Interstitial murine cytomegalovirus pneumonia after irradiation: characterization of cells that limit viral replication during established infection of the lungs. J Virol. 1985;55: 264–273. doi: 10.1128/JVI.55.2.264-273.1985 2991554 PMC254929

[31] Steffens HP, Kurz S, Holtappels R, Reddehase MJ. Preemptive CD8 T-cell immunotherapy of acute cytomegalovirus infection prevents lethal disease, limits the burden of latent viral genomes, and reduces the risk of virus recurrence. J Virol. 1998;72: 1797–1804. doi: 10.1128/JVI.72.3.1797-1804.1998 9499030 PMC109469

[32] Thomas S, Klobuch S, Podlech J, Plachter B, Hoffmann P, Renzaho A, et al. Evaluating human T-cell therapy of cytomegalovirus organ disease in HLA-transgenic mice. PLoS Pathog. 2015;11: e1005049. doi: 10.1371/journal.ppat.1005049 26181057 PMC4504510

[33] Renzaho A, Podlech J, Kühnapfel B, Blaum F, Reddehase MJ, Lemmermann NAW. Cytomegalovirus-associated inhibition of hematopoiesis is preventable by cytoimmunotherapy with antiviral CD8 T cells. Front Cell Infect Microbiol. 2020;10: 138. doi: 10.3389/fcimb.2020.00138 32373544 PMC7186302

[34] Gergely KM, Podlech J, Becker S, Freitag K, Krauter S, Büscher N, et al. Therapeutic vaccination of hematopoietic cell transplantation recipients improves protective CD8 T-cell immunotherapy of cytomegalovirus infection. Front Immunol. 2021;12: 694588. doi: 10.3389/fimmu.2021.694588 34489940 PMC8416627

[35] Riddell SR, Watanabe KS, Goodrich JM, Li CR, Agha ME, Greenberg PD. Restoration of viral immunity in immunodeficient humans by the adoptive transfer of T cell clones. Science. 1992;257: 238–241. doi: 10.1126/science.1352912 1352912

[36] Walter EA, Greenberg PD, Gilbert MJ, Finch RJ, Watanabe KS, Thomas ED, et al. Reconstitution of cellular immunity against cytomegalovirus in recipients of allogeneic bone marrow by transfer of T-cell clones from the donor. N Engl J Med. 1995;333: 1038–1044. doi: 10.1056/NEJM199510193331603 7675046

[37] Peggs KS, Verfuerth S, Pizzey A, Chow S-LC, Thomson K, Mackinnon S. Cytomegalovirus-specific T cell immunotherapy promotes restoration of durable functional antiviral immunity following allogeneic stem cell transplantation. Clin Infect Dis. 2009;49: 1851–1860. doi: 10.1086/648422 19911966

[38] Schmitt A, Tonn T, Busch DH, Grigoleit GU, Einsele H, Odendahl M, et al. Adoptive transfer and selective reconstitution of streptamer-selected cytomegalovirus-specific CD8+ T cells leads to virus clearance in patients after allogeneic peripheral blood stem cell transplantation. Transfusion. 2011;51: 591–599. doi: 10.1111/j.1537-2995.2010.02940.x 21133926

[39] Stemberger C, Graef P, Odendahl M, Albrecht J, Dössinger G, Anderl F, et al. Lowest numbers of primary CD8(+) T cells can reconstitute protective immunity upon adoptive immunotherapy. Blood. 2014;124: 628–637. doi: 10.1182/blood-2013-12-547349 24855206

[40] Einsele H, Roosnek E, Rufer N, Sinzger C, Riegler S, Löffler J, et al. Infusion of cytomegalovirus (CMV)-specific T cells for the treatment of CMV infection not responding to antiviral chemotherapy. Blood. 2002;99: 3916–3922. doi: 10.1182/blood.v99.11.3916 12010789

[41] Feuchtinger T, Opherk K, Bethge WA, Topp MS, Schuster FR, Weissinger EM, et al. Adoptive transfer of pp65-specific T cells for the treatment of chemorefractory cytomegalovirus disease or reactivation after haploidentical and matched unrelated stem cell transplantation. Blood. 2010;116: 4360–4367. doi: 10.1182/blood-2010-01-262089 20625005

[42] Bao L, Cowan MJ, Dunham K, Horn B, McGuirk J, Gilman A, et al. Adoptive immunotherapy with CMV-specific cytotoxic T lymphocytes for stem cell transplant patients with refractory CMV infections. J Immunother. 2012;35: 293–298. doi: 10.1097/CJI.0b013e31824300a2 22421947 PMC3306600

[43] Odendahl M, Grigoleit GU, Bönig H, Neuenhahn M, Albrecht J, Anderl F, et al. Clinical-scale isolation of “minimally manipulated” cytomegalovirus-specific donor lymphocytes for the treatment of refractory cytomegalovirus disease. Cytotherapy. 2014;16: 1245–1256. doi: 10.1016/j.jcyt.2014.05.023 25108651

[44] Pei X-Y, Zhao X-Y, Chang Y-J, Liu J, Xu L-P, Wang Y, et al. Cytomegalovirus-specific T-cell transfer for refractory cytomegalovirus infection after haploidentical stem cell transplantation: the quantitative and qualitative immune recovery for cytomegalovirus. J Infect Dis. 2017;216: 945–956. doi: 10.1093/infdis/jix357 29029297

[45] Chemaly RF, Chou S, Einsele H, Griffiths P, Avery R, Razonable RR, et al. Definitions of resistant and refractory cytomegalovirus infection and disease in transplant recipients for use in clinical trials. Clin Infect Dis. 2019;68: 1420–1426. doi: 10.1093/cid/ciy696 30137245 PMC7348585

[46] Kaeuferle T, Krauss R, Blaeschke F, Willier S, Feuchtinger T. Strategies of adoptive T -cell transfer to treat refractory viral infections post allogeneic stem cell transplantation. J Hematol Oncol. 2019;12: 13. doi: 10.1186/s13045-019-0701-1 30728058 PMC6364410

[47] Reddehase MJ, Jonjić S, Weiland F, Mutter W, Koszinowski UH. Adoptive immunotherapy of murine cytomegalovirus adrenalitis in the immunocompromised host: CD4-helper-independent antiviral function of CD8-positive memory T lymphocytes derived from latently infected donors. J Virol. 1988;62: 1061–1065. doi: 10.1128/JVI.62.3.1061-1065.1988 2828654 PMC253668

[48] Pahl-Seibert M-F, Juelch M, Podlech J, Thomas D, Deegen P, Reddehase MJ, et al. Highly protective in vivo function of cytomegalovirus IE1 epitope-specific memory CD8 T cells purified by T-cell receptor-based cell sorting. J Virol. 2005;79: 5400–5413. doi: 10.1128/JVI.79.9.5400-5413.2005 15827154 PMC1082747

[49] Böhm V, Podlech J, Thomas D, Deegen P, Pahl-Seibert M-F, Lemmermann NAW, et al. Epitope-specific in vivo protection against cytomegalovirus disease by CD8 T cells in the murine model of preemptive immunotherapy. Med Microbiol Immunol. 2008;197: 135–144. doi: 10.1007/s00430-008-0092-3 18340461

[50] Ebert S, Podlech J, Gillert-Marien D, Gergely KM, Büttner JK, Fink A, et al. Parameters determining the efficacy of adoptive CD8 T-cell therapy of cytomegalovirus infection. Med Microbiol Immunol. 2012;201: 527–539. doi: 10.1007/s00430-012-0258-x 22972232

[51] Cobbold M, Khan N, Pourgheysari B, Tauro S, McDonald D, Osman H, et al. Adoptive transfer of cytomegalovirus-specific CTL to stem cell transplant patients after selection by HLA-peptide tetramers. J Exp Med. 2005;202: 379–386. doi: 10.1084/jem.20040613 16061727 PMC2213070

[52] Peggs KS, Verfuerth S, Pizzey A, Khan N, Guiver M, Moss PA, et al. Adoptive cellular therapy for early cytomegalovirus infection after allogeneic stem-cell transplantation with virus-specific T-cell lines. Lancet. 2003;362: 1375–1377. doi: 10.1016/S0140-6736(03)14634-X 14585640

[53] Holtappels R, Freitag K, Renzaho A, Becker S, Lemmermann NAW, Reddehase MJ. Revisiting CD8 T-cell “memory inflation”: new insights with implications for cytomegaloviruses as vaccine vectors. Vaccines (Basel). 2020;8: 402. doi: 10.3390/vaccines8030402 32707744 PMC7563500

[54] Snyder CM, Cho KS, Bonnett EL, van Dommelen S, Shellam GR, Hill AB. Memory inflation during chronic viral infection is maintained by continuous production of short-lived, functional T cells. Immunity. 2008;29: 650–659. doi: 10.1016/j.immuni.2008.07.017 18957267 PMC2583440

[55] Baumann NS, Torti N, Welten SPM, Barnstorf I, Borsa M, Pallmer K, et al. Tissue maintenance of CMV-specific inflationary memory T cells by IL-15. PLoS Pathog. 2018;14: e1006993. doi: 10.1371/journal.ppat.1006993 29652930 PMC5919076

[56] Seckert CK, Griessl M, Büttner JK, Scheller S, Simon CO, Kropp KA, et al. Viral latency drives “memory inflation”: a unifying hypothesis linking two hallmarks of cytomegalovirus infection. Med Microbiol Immunol. 2012;201: 551–566. doi: 10.1007/s00430-012-0273-y 22991040

[57] Klenerman P, Oxenius A. T cell responses to cytomegalovirus. Nat Rev Immunol. 2016;16: 367–377. doi: 10.1038/nri.2016.38 27108521

[58] Cicin-Sain L. Cytomegalovirus memory inflation and immune protection. Med Microbiol Immunol. 2019;208: 339–347. doi: 10.1007/s00430-019-00607-8 30972476

[59] Welten SPM, Baumann NS, Oxenius A. Fuel and brake of memory T cell inflation. Med Microbiol Immunol. 2019;208: 329–338. doi: 10.1007/s00430-019-00587-9 30852648

[60] Kurz SK, Rapp M, Steffens HP, Grzimek NK, Schmalz S, Reddehase MJ. Focal transcriptional activity of murine cytomegalovirus during latency in the lungs. J Virol. 1999;73: 482–494. doi: 10.1128/JVI.73.1.482-494.1999 9847354 PMC103855

[61] Simon CO, Holtappels R, Tervo H-M, Böhm V, Däubner T, Oehrlein-Karpi SA, et al. CD8 T cells control cytomegalovirus latency by epitope-specific sensing of transcriptional reactivation. J Virol. 2006;80: 10436–10456. doi: 10.1128/JVI.01248-06 16928768 PMC1641801

[62] Griessl M, Renzaho A, Freitag K, Seckert CK, Reddehase MJ, Lemmermann NAW. Stochastic episodes of latent cytomegalovirus transcription drive CD8 T-cell “memory inflation” and avoid Immune evasion. Front Immunol. 2021;12: 668885. doi: 10.3389/fimmu.2021.668885 33968074 PMC8100209

[63] Seckert CK, Renzaho A, Tervo H-M, Krause C, Deegen P, Kühnapfel B, et al. Liver sinusoidal endothelial cells are a site of murine cytomegalovirus latency and reactivation. J Virol. 2009;83: 8869–8884. doi: 10.1128/JVI.00870-09 19535440 PMC2738169

[64] Munks MW, Rott K, Nesterenko PA, Smart SM, Williams V, Tatum A, et al. Latent CMV infection of lymphatic endothelial cells is sufficient to drive CD8 T cell memory inflation. PLoS Pathog. 2023;19: e1010351. doi: 10.1371/journal.ppat.1010351 36689486 PMC9894547

[65] Sitnik KM, Krstanović F, Gödecke N, Rand U, Kubsch T, Maaß H, et al. Fibroblasts are a site of murine cytomegalovirus lytic replication and Stat1-dependent latent persistence in vivo. Nat Commun. 2023;14: 3087. doi: 10.1038/s41467-023-38449-x 37248241 PMC10227055

[66] Moss P, Rickinson A. Cellular immunotherapy for viral infection after HSC transplantation. Nat Rev Immunol. 2005;5: 9–20. doi: 10.1038/nri1526 15630425

[67] Adler B, Sinzger C. Cytomegalovirus interstrain variance in cell type tropism. In: Reddehase MJ, editor. Cytomegaloviruses: from molecular pathogenesis to intervention. Norfolk: Caister academic press; 2013. pp. 297–321.

[68] Wilkinson GWG, Davison AJ, Tomasec P, Fielding CA, Aicheler R, Murrell I, et al. Human cytomegalovirus: taking the strain. Med Microbiol Immunol. 2015;204: 273–284. doi: 10.1007/s00430-015-0411-4 25894764 PMC4439430

[69] Wang H-Y, Valencia SM, Pfeifer SP, Jensen JD, Kowalik TF, Permar SR. Common polymorphisms in the glycoproteins of human cytomegalovirus and associated strain-specific immunity. Viruses. 2021;13: 1106. doi: 10.3390/v13061106 34207868 PMC8227702

[70] Holtappels R, Böhm V, Podlech J, Reddehase MJ. CD8 T-cell-based immunotherapy of cytomegalovirus infection: “proof of concept” provided by the murine model. Med Microbiol Immunol. 2008;197: 125–134. doi: 10.1007/s00430-008-0093-2 18343947

[71] Holtappels R, Ebert S, Podlech J, Fink A, Böhm V, Lemmermann NAW, et al. Murine model for cytoimmunotherapy of CMV disease after haematopoietic cell transplantation. In: Reddehase MJ, editor. Cytomegaloviruses: from molecular pathogenesis to intervention. Norfolk: Caister academic press; 2013. pp. 352–379.

[72] Reddehase MJ, Balthesen M, Rapp M, Jonjić S, Pavić I, Koszinowski UH. The conditions of primary infection define the load of latent viral genome in organs and the risk of recurrent cytomegalovirus disease. J Exp Med. 1994;179: 185–193. doi: 10.1084/jem.179.1.185 8270864 PMC2191331

[73] Kurz SK, Reddehase MJ. Patchwork pattern of transcriptional reactivation in the lungs indicates sequential checkpoints in the transition from murine cytomegalovirus latency to recurrence. J Virol. 1999;73: 8612–8622. doi: 10.1128/JVI.73.10.8612-8622.1999 10482614 PMC112881

[74] Welsh RM, Selin LK. No one is naive: the significance of heterologous T-cell immunity. Nat Rev Immunol. 2002;2: 417–426. doi: 10.1038/nri820 12093008

[75] Holtappels R, Thomas D, Podlech J, Reddehase MJ. Two antigenic peptides from genes m123 and m164 of murine cytomegalovirus quantitatively dominate CD8 T-cell memory in the H-2d haplotype. J Virol. 2002;76: 151–164. doi: 10.1128/jvi.76.1.151-164.2002 11739681 PMC135724

[76] Munks MW, Gold MC, Zajac AL, Doom CM, Morello CS, Spector DH, et al. Genome-wide analysis reveals a highly diverse CD8 T cell response to murine cytomegalovirus. J Immunol. 2006;176: 3760–3766. doi: 10.4049/jimmunol.176.6.3760 16517745

[77] Seckert CK, Schader SI, Ebert S, Thomas D, Freitag K, Renzaho A, et al. Antigen-presenting cells of haematopoietic origin prime cytomegalovirus-specific CD8 T-cells but are not sufficient for driving memory inflation during viral latency. J Gen Virol. 2011;92: 1994–2005. doi: 10.1099/vir.0.031815-0 21632567

[78] Reddehase MJ, Mutter W, Münch K, Bühring HJ, Koszinowski UH. CD8-positive T lymphocytes specific for murine cytomegalovirus immediate-early antigens mediate protective immunity. J Virol. 1987;61: 3102–3108. doi: 10.1128/JVI.61.10.3102-3108.1987 3041033 PMC255886

[79] Lemmermann NAW, Kropp KA, Seckert CK, Grzimek NKA, Reddehase MJ. Reverse genetics modification of cytomegalovirus antigenicity and immunogenicity by CD8 T-cell epitope deletion and insertion. J Biomed Biotechnol. 2011;2011: 812742. doi: 10.1155/2011/812742 21253509 PMC3021883

[80] Sacher T, Podlech J, Mohr CA, Jordan S, Ruzsics Z, Reddehase MJ, et al. The major virus-producing cell type during murine cytomegalovirus infection, the hepatocyte, is not the source of virus dissemination in the host. Cell Host Microbe. 2008;3: 263–272. doi: 10.1016/j.chom.2008.02.014 18407069

[81] Lemmermann NAW, Krmpotic A, Podlech J, Brizic I, Prager A, Adler H, et al. Non-redundant and redundant roles of cytomegalovirus gH/gL complexes in host organ entry and intra-tissue spread. PLoS Pathog. 2015;11: e1004640. doi: 10.1371/journal.ppat.1004640 25659098 PMC4450070

[82] Reddehase MJ, Rothbard JB, Koszinowski UH. A pentapeptide as minimal antigenic determinant for MHC class I-restricted T lymphocytes. Nature. 1989;337: 651–653. doi: 10.1038/337651a0 2465495

[83] Reddehase MJ, Koszinowski UH. Redistribution of critical major histocompatibility complex and T cell receptor-binding functions of residues in an antigenic sequence after biterminal substitution. Eur J Immunol. 1991;21: 1697–1701. doi: 10.1002/eji.1830210717 2060579

[84] Holzhütter HG, Frömmel C, Kloetzel PM. A theoretical approach towards the identification of cleavage-determining amino acid motifs of the 20 S proteasome. J Mol Biol. 1999;286: 1251–1265. doi: 10.1006/jmbi.1998.2530 10047495

[85] Tenzer S, Peters B, Bulik S, Schoor O, Lemmel C, Schatz MM, et al. Modeling the MHC class I pathway by combining predictions of proteasomal cleavage, TAP transport and MHC class I binding. Cell Mol Life Sci. 2005;62: 1025–1037. doi: 10.1007/s00018-005-4528-2 15868101 PMC11924537

[86] Xie J, Xu Z, Zhou S, Pan X, Cai S, Yang L, et al. The VHSE-based prediction of proteasomal cleavage sites. PLoS One. 2013;8: e74506. doi: 10.1371/journal.pone.0074506 24040264 PMC3767653

[87] Thomas C, Tampé R. MHC I assembly and peptide editing—chaperones, clients, and molecular plasticity in immunity. Curr Opin Immunol. 2021;70: 48–56. doi: 10.1016/j.coi.2021.02.004 33689959

[88] Margulies DH, Taylor DK, Jiang J, Boyd LF, Ahmad J, Mage MG, et al. Chaperones and catalysts: how antigen presentation pathways cope with biological necessity. Front Immunol. 2022;13: 859782. doi: 10.3389/fimmu.2022.859782 35464465 PMC9022212

[89] Holtappels R, Podlech J, Freitag K, Lemmermann NA, Reddehase MJ. Memory CD8 T cells protect against cytomegalovirus disease by formation of nodular inflammatory foci preventing intra-tissue virus spread. Viruses. 2022;14: 1145. doi: 10.3390/v14061145 35746617 PMC9229300

[90] Nauerth M, Weißbrich B, Knall R, Franz T, Dössinger G, Bet J, et al. TCR-ligand koff rate correlates with the protective capacity of antigen-specific CD8+ T cells for adoptive transfer. Sci Transl Med. 2013;5: 192ra87. doi: 10.1126/scitranslmed.3005958 23825303 PMC3991308

[91] Hamdan S, Reddehase MJ, Holtappels R. Cytomegalovirus immune evasion sets the functional avidity threshold for protection by CD8 T cells. Med Microbiol Immunol. 2023;212: 153–163. doi: 10.1007/s00430-022-00733-w 35364731 PMC10085950

[92] Lyons AB, Parish CR. Determination of lymphocyte division by flow cytometry. J Immunol Methods. 1994;171: 131–137. doi: 10.1016/0022-1759(94)90236-4 8176234

[93] Quah BJC, Warren HS, Parish CR. Monitoring lymphocyte proliferation in vitro and in vivo with the intracellular fluorescent dye carboxyfluorescein diacetate succinimidyl ester. Nat Protoc. 2007;2: 2049–2056. doi: 10.1038/nprot.2007.296 17853860

[94] Celis JE, Madsen P, Celis A, Nielsen HV, Gesser B. Cyclin (PCNA, auxiliary protein of DNA polymerase delta) is a central component of the pathway(s) leading to DNA replication and cell division. FEBS Lett. 1987;220: 1–7. doi: 10.1016/0014-5793(87)80865-7 2886367

[95] Kurki P, Ogata K, Tan EM. Monoclonal antibodies to proliferating cell nuclear antigen (PCNA)/cyclin as probes for proliferating cells by immunofluorescence microscopy and flow cytometry. J Immunol Methods. 1988;109: 49–59. doi: 10.1016/0022-1759(88)90441-3 2895795

[96] Bologna-Molina R, Mosqueda-Taylor A, Molina-Frechero N, Mori-Estevez A-D, Sánchez-Acuña G. Comparison of the value of PCNA and Ki-67 as markers of cell proliferation in ameloblastic tumors. Med Oral Patol Oral Cir Bucal. 2013;18: e174–179. doi: 10.4317/medoral.18573 23229269 PMC3613329

[97] Hall PA, Levison DA, Woods AL, Yu CC, Kellock DB, Watkins JA, et al. Proliferating cell nuclear antigen (PCNA) immunolocalization in paraffin sections: an index of cell proliferation with evidence of deregulated expression in some neoplasms. J Pathol. 1990;162: 285–294. doi: 10.1002/path.1711620403 1981239

[98] Mate JL, Ariza A, Muñoz A, Molinero JL, López D, Navas-Palacios JJ. Induction of proliferating cell nuclear antigen and Ki-67 expression by cytomegalovirus infection. J Pathol. 1998;184: 279–282. doi: 10.1002/(SICI)1096-9896(199803)184:3&lt;279::AID-PATH7&gt;3.0.CO;2-4 9614380

[99] Reddehase MJ, Lemmermann NAW. Cellular reservoirs of latent cytomegaloviruses. Med Microbiol Immunol. 2019;208: 391–403. doi: 10.1007/s00430-019-00592-y 31011793

[100] McLaughlin-Taylor E, Pande H, Forman SJ, Tanamachi B, Li CR, Zaia JA, et al. Identification of the major late human cytomegalovirus matrix protein pp65 as a target antigen for CD8+ virus-specific cytotoxic T lymphocytes. J Med Virol. 1994;43: 103–110. doi: 10.1002/jmv.1890430119 8083644

[101] Boppana SB, Britt WJ. Recognition of human cytomegalovirus gene products by HCMV-specific cytotoxic T cells. Virology. 1996;222: 293–296. doi: 10.1006/viro.1996.0424 8806513

[102] Wills MR, Carmichael AJ, Mynard K, Jin X, Weekes MP, Plachter B, et al. The human cytotoxic T-lymphocyte (CTL) response to cytomegalovirus is dominated by structural protein pp65: frequency, specificity, and T-cell receptor usage of pp65-specific CTL. J Virol. 1996;70: 7569–7579. doi: 10.1128/JVI.70.11.7569-7579.1996 8892876 PMC190825

[103] Weekes MP, Wills MR, Mynard K, Carmichael AJ, Sissons JG. The memory cytotoxic T-lymphocyte (CTL) response to human cytomegalovirus infection contains individual peptide-specific CTL clones that have undergone extensive expansion in vivo. J Virol. 1999;73: 2099–2108. doi: 10.1128/JVI.73.3.2099-2108.1999 9971792 PMC104454

[104] Sylwester AW, Mitchell BL, Edgar JB, Taormina C, Pelte C, Ruchti F, et al. Broadly targeted human cytomegalovirus-specific CD4+ and CD8+ T cells dominate the memory compartments of exposed subjects. J Exp Med. 2005;202: 673–685. doi: 10.1084/jem.20050882 16147978 PMC2212883

[105] Graef P, Buchholz VR, Stemberger C, Flossdorf M, Henkel L, Schiemann M, et al. Serial transfer of single-cell-derived immunocompetence reveals stemness of CD8(+) central memory T cells. Immunity. 2014;41: 116–126. doi: 10.1016/j.immuni.2014.05.018 25035956

[106] Poole E, Sinclair J. Sleepless latency of human cytomegalovirus. Med Microbiol Immunol. 2015;204: 421–429. doi: 10.1007/s00430-015-0401-6 25772624 PMC4439429

[107] Collins-McMillen D, Goodrum FD. The loss of binary: pushing the herpesvirus latency paradigm. Curr Clin Microbiol Rep. 2017;4: 124–131. doi: 10.1007/s40588-017-0072-8 29250481 PMC5726573

[108] Shnayder M, Nachshon A, Krishna B, Poole E, Boshkov A, Binyamin A, et al. Defining the transcriptional landscape during cytomegalovirus latency with single-cell RNA sequencing. mBio. 2018;9: e00013–18. doi: 10.1128/mBio.00013-18 29535194 PMC5850328

[109] Schwartz M, Stern-Ginossar N. The transcriptome of latent human cytomegalovirus. J Virol. 2019;93: e00047–19. doi: 10.1128/JVI.00047-19 30867313 PMC6532091

[110] Smith CJ, Venturi V, Quigley MF, Turula H, Gostick E, Ladell K, et al. Stochastic expansions maintain the clonal stability of CD8+ T cell populations undergoing memory inflation driven by murine cytomegalovirus. J Immunol. 2020;204: 112–121. doi: 10.4049/jimmunol.1900455 31818981 PMC6920548

[111] Redeker A, Welten SPM, Arens R. Viral inoculum dose impacts memory T-cell inflation. Eur J Immunol. 2014;44: 1046–1057. doi: 10.1002/eji.201343946 24356925

[112] Adler SP, Reddehase MJ. Pediatric roots of cytomegalovirus recurrence and memory inflation in the elderly. Med Microbiol Immunol. 2019;208: 323–328. doi: 10.1007/s00430-019-00609-6 31062089

[113] Jackson SE, Sedikides GX, Okecha G, Wills MR. Generation, maintenance and tissue distribution of T cell responses to human cytomegalovirus in lytic and latent infection. Med Microbiol Immunol. 2019;208: 375–389. doi: 10.1007/s00430-019-00598-6 30895366 PMC6647459

[114] Reddehase MJ. “Checks and balances” in cytomegalovirus-host cohabitation. Med Microbiol Immunol. 2019;208: 259–261. doi: 10.1007/s00430-019-00618-5 31129788

[115] van den Berg SPH, Pardieck IN, Lanfermeijer J, Sauce D, Klenerman P, van Baarle D, et al. The hallmarks of CMV-specific CD8 T-cell differentiation. Med Microbiol Immunol. 2019;208: 365–373. doi: 10.1007/s00430-019-00608-7 30989333 PMC6647465

[116] Klenerman P. The (gradual) rise of memory inflation. Immunol Rev. 2018;283: 99–112. doi: 10.1111/imr.12653 29664577 PMC5947157

[117] Holtappels R, Pahl-Seibert MF, Thomas D, Reddehase MJ. Enrichment of immediate-early 1 (m123/pp89) peptide-specific CD8 T cells in a pulmonary CD62L(lo) memory-effector cell pool during latent murine cytomegalovirus infection of the lungs. J Virol. 2000;74: 11495–11503. doi: 10.1128/jvi.74.24.11495-11503.2000 11090146 PMC112429

[118] Simmons P, Kaushansky K, Torok-Storb B. Mechanisms of cytomegalovirus-mediated myelosuppression: perturbation of stromal cell function versus direct infection of myeloid cells. Proc Natl Acad Sci U S A. 1990;87: 1386–1390. doi: 10.1073/pnas.87.4.1386 2154745 PMC53480

[119] Pignatelli S, Dal Monte P, Rossini G, Landini MP. Genetic polymorphisms among human cytomegalovirus (HCMV) wild-type strains. Rev Med Virol. 2004;14: 383–410. doi: 10.1002/rmv.438 15386592

[120] Suárez NM, Wilkie GS, Hage E, Camiolo S, Holton M, Hughes J, et al. Human cytomegalovirus genomes sequenced directly from clinical material: variation, multiple-strain infection, recombination, and gene loss. J Infect Dis. 2019;220: 781–791. doi: 10.1093/infdis/jiz208 31050742 PMC6667795

[121] Charles OJ, Venturini C, Gantt S, Atkinson C, Griffiths P, Goldstein RA, et al. Genomic and geographical structure of human cytomegalovirus. Proc Natl Acad Sci U S A. 2023;120: e2221797120. doi: 10.1073/pnas.2221797120 37459519 PMC10372631

[122] Holtappels R, Podlech J, Geginat G, Steffens HP, Thomas D, Reddehase MJ. Control of murine cytomegalovirus in the lungs: relative but not absolute immunodominance of the immediate-early 1 nonapeptide during the antiviral cytolytic T-lymphocyte response in pulmonary infiltrates. J Virol. 1998;72: 7201–7212. doi: 10.1128/JVI.72.9.7201-7212.1998 9696814 PMC109942

[123] Masopust D, Vezys V, Usherwood EJ, Cauley LS, Olson S, Marzo AL, et al. Activated primary and memory CD8 T cells migrate to nonlymphoid tissues regardless of site of activation or tissue of origin. J Immunol. 2004;172: 4875–4882. doi: 10.4049/jimmunol.172.8.4875 15067066

[124] Marzo AL, Yagita H, Lefrançois L. Cutting edge: migration to nonlymphoid tissues results in functional conversion of central to effector memory CD8 T cells. J Immunol. 2007;179: 36–40. doi: 10.4049/jimmunol.179.1.36 17579018 PMC2861291

[125] Blaum F, Lukas D, Reddehase MJ, Lemmermann NAW. Localization of viral epitope-specific CD8 T cells during cytomegalovirus latency in the lungs and recruitment to lung parenchyma by airway challenge infection. Life (Basel). 2021;11: 918. doi: 10.3390/life11090918 34575067 PMC8467276

[126] Podlech J, Holtappels R, Grzimek NKA, Reddehase MJ. Animal models: murine cytomegalovirus. 2nd ed. In: Kabelitz D, Kaufmann S, editors. Methods in Microbiology: Immunology of Infection. 2nd ed. Academic Press London; 2002. pp. 493–525.

[127] Lemmermann NAW, Podlech J, Seckert CK, Kropp KA, Grzimek NKA, Reddehase MJ, et al. CD8 T-cell immunotherapy of cytomegalovirus disease in the murine model. 3rd ed. In: Kabelitz D, Kaufmann S, editors. Methods in Microbiology: Immunology of Infection. 3rd ed. Academic Press London; 2010. pp. 369–420. doi: 10.1016/S0580-9517(10)37016-4

[128] Böhm V, Simon CO, Podlech J, Seckert CK, Gendig D, Deegen P, et al. The immune evasion paradox: immunoevasins of murine cytomegalovirus enhance priming of CD8 T cells by preventing negative feedback regulation. J Virol. 2008;82: 11637–11650. doi: 10.1128/JVI.01510-08 18815306 PMC2583666

[129] Freitag K, Hamdan S, Reddehase MJ, Holtappels R. Immunodominant cytomegalovirus epitopes suppress subdominant epitopes in the generation of high-avidity CD8 T cells. Pathogens. 2021;10:956. doi: 10.3390/pathogens10080956 34451420 PMC8400798

