# Supplementary figures and images for: Immunotherapy of cytomegalovirus infection by low-dose adoptive transfer of antiviral CD8 T cells relies on substantial post-transfer expansion of central memory cells but not effector-memory cells
Source: PLoS Pathog. 2023 Nov 16;19(11):e1011643. doi: 10.1371/journal.ppat.1011643 (PMC10688903; doi:10.1371/journal.ppat.1011643)

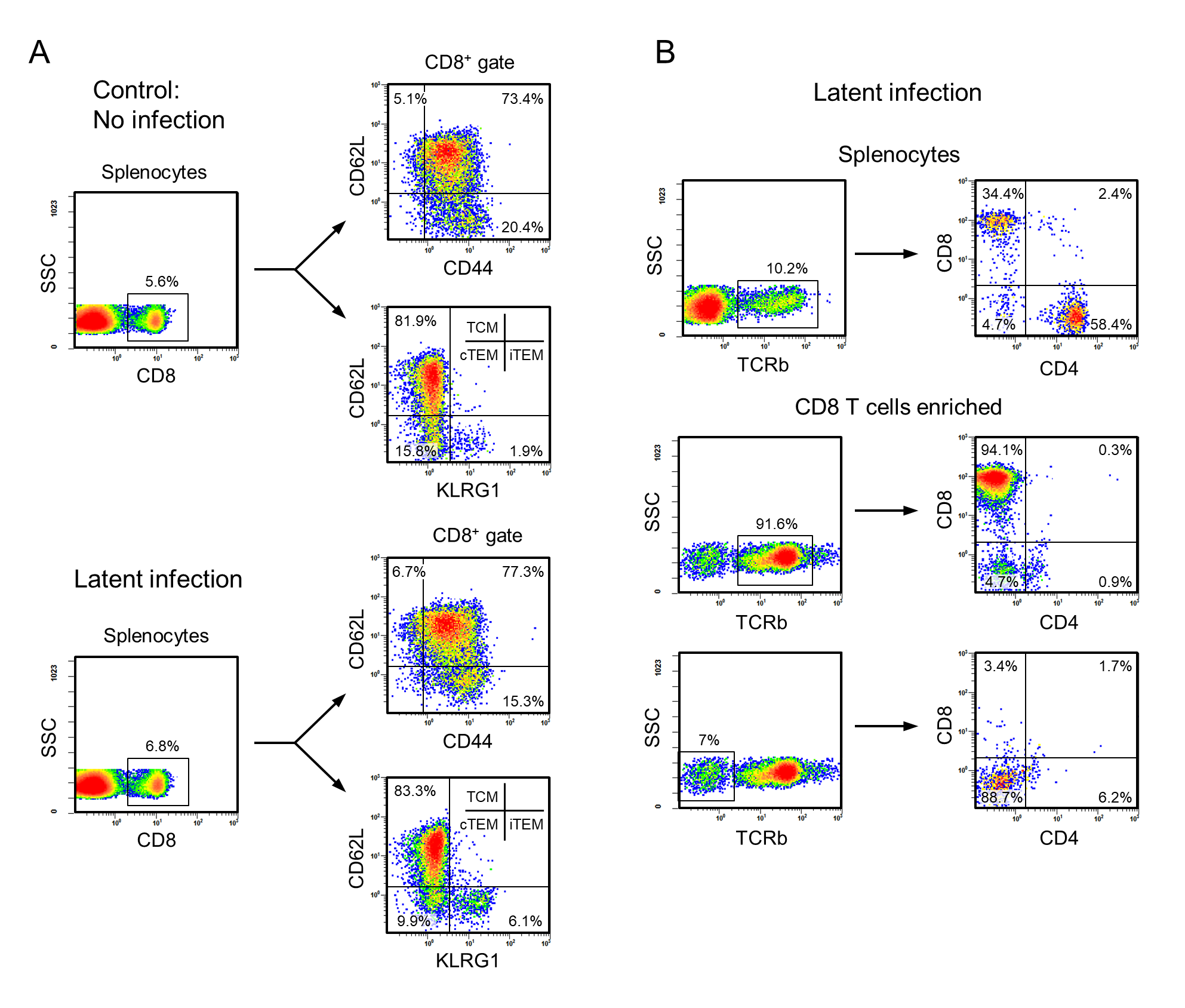

Supplement: S1 Fig — (A) CFM analyses of cell surface marker expression by CD8 T cells derived from the spleen of age-matched uninfected BALB/c mice (upper panels) and of memory CD8 T cells derived from the spleen of BALB/c AT donor mice in the stage of latent infection at 8 months after priming by infection with mCMV-WT (lower panels). Shown are color-coded 2D fluorescence density plots for the cell surface marker combinations indicated, with red and blue color representing highest and lowest cell numbers, respectively. Gates were set on CD8 T cells in the SSC (sideward scatter) versus CD8 plots. (TCM) T central memory cells. (cTEM) conventional T effector-memory cells. (iTEM) inflationary T effector-memory cells. (B) CFM analyses documenting the successful enrichment of CD8 T cells by immunomagnetic cell sorting. (TIF) [file ppat.1011643.s001.TIF]

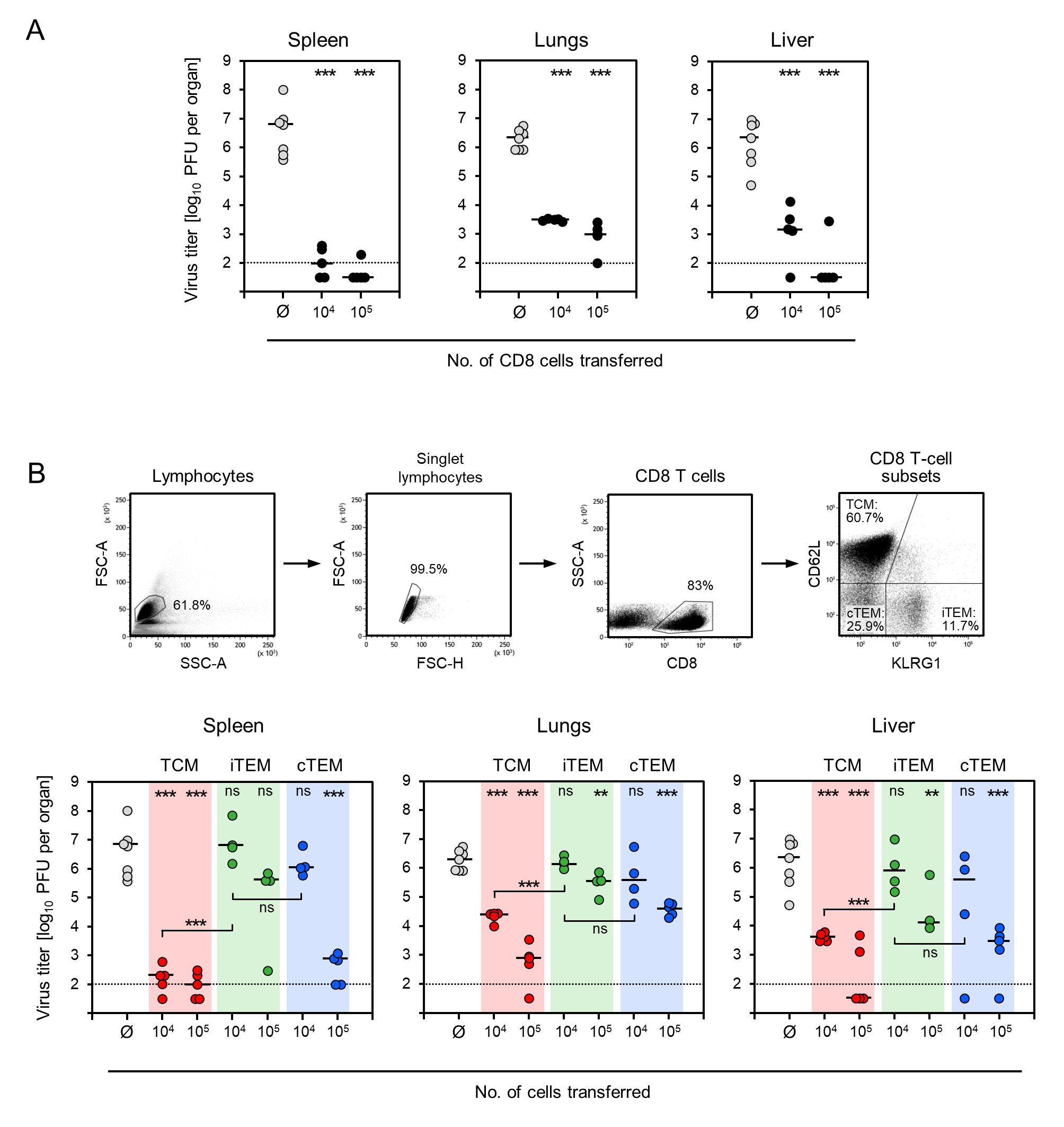

Supplement: S2 Fig — (A) AT of unseparated memory CD8 T cells. For details, see the Legend of Fig 1C in the body of the text. (B) AT of memory CD8 T-cell subsets. (TCM) T central memory cells. (cTEM) conventional T effector-memory cells. (iTEM) inflationary T effector-memory cells. For details, see the Legend of Fig 3B in the body of the text. Asterisk-coded statistical significance levels for differences between the AT groups and the no-AT control group (Ø): (**) P< 0.01, and (***) P< 0.001. (ns) not significant. (TIF) [file ppat.1011643.s002.TIF]

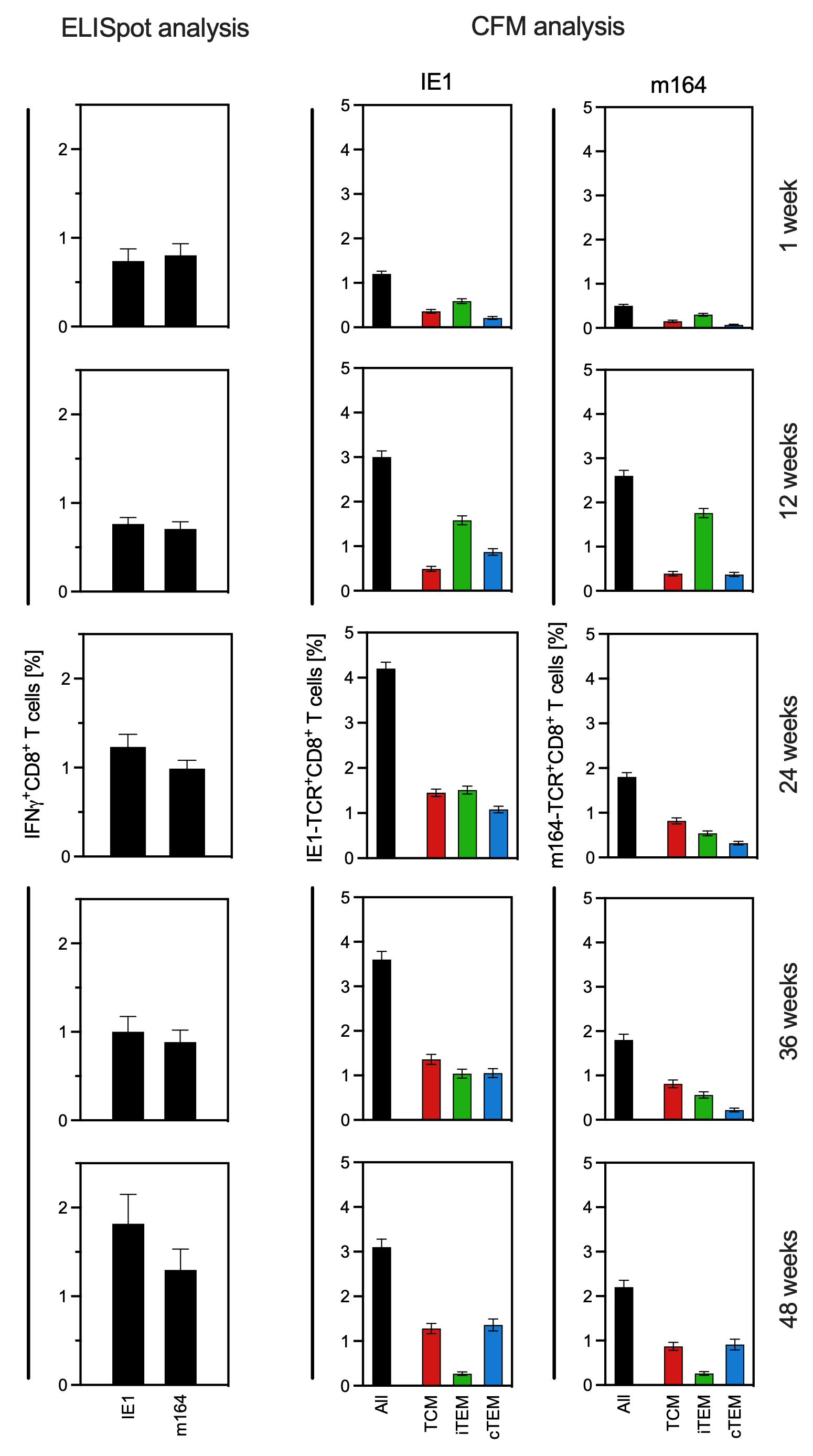

Supplement: S3 Fig — Data result from a reanalysis of a previously published experiment [53] with a different focus of interpretation. BALB/c AT donor mice were infected with mCMV-WT, and immunomagnetically enriched spleen-derived memory CD8 T cells were tested at the times indicated. (Left column) Frequencies of functional IE1 and m164 epitope-specific cells among total memory CD8 T cells determined by the ELISpot assay. (Right colums) Frequencies of memory CD8 T cells, total or differentiated by activation subset, expressing IE1 and m164 epitope-specific TCRs detected by CFM analysis (for the method, see [53]). (TCM) T central memory cells. (cTEM) conventional T effector-memory cells. (iTEM) inflationary T effector-memory cells. Error bars are indicated. (TIF) [file ppat.1011643.s003.tif]

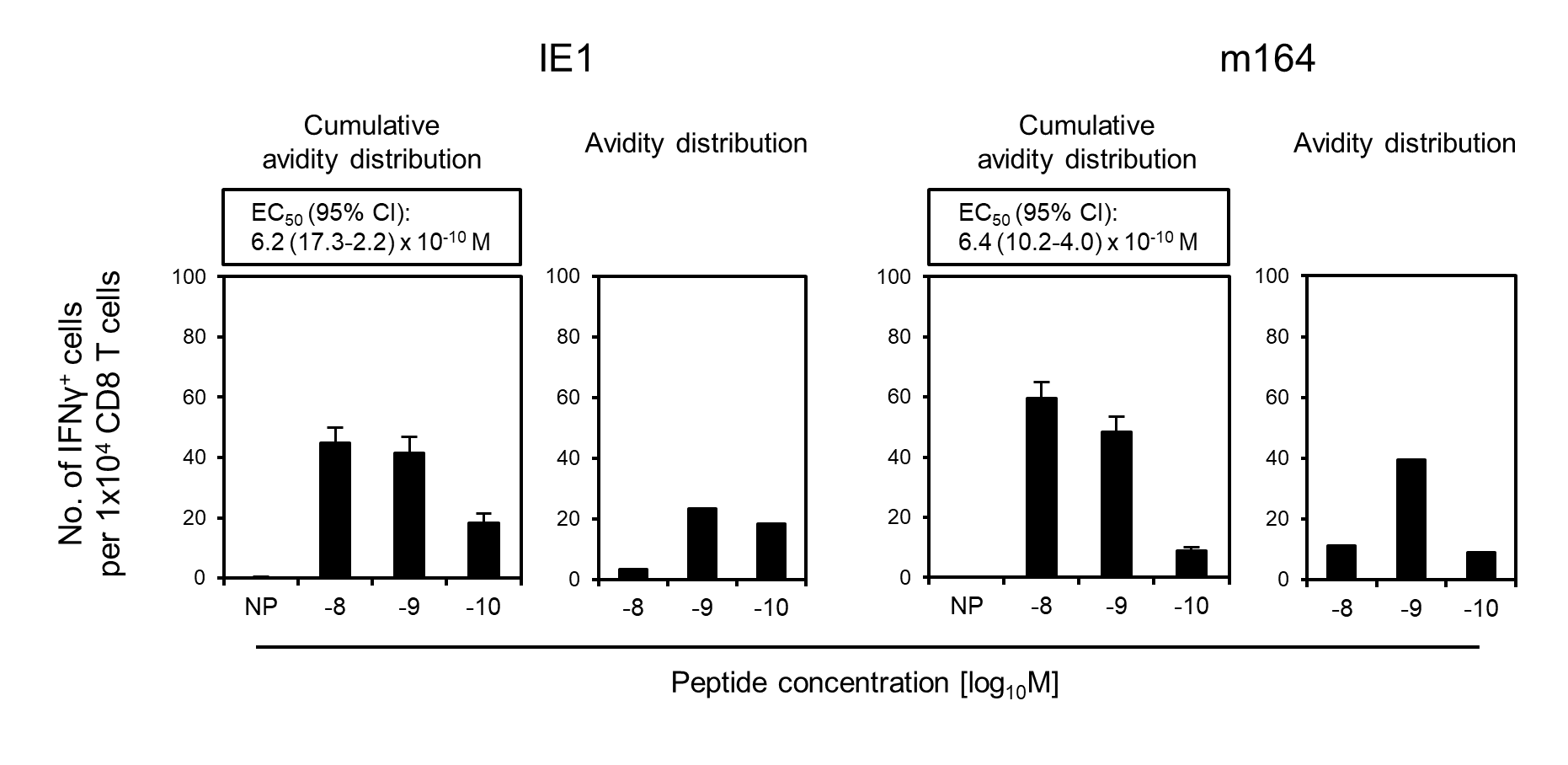

Supplement: S4 Fig — Shown are cumulative avidity distributions and deduced Gaussian-like avidity distributions of unseparated memory CD8 T cells specific for antigenic peptides IE1 and m164. For details, see the Legend of Fig 4 in the body of the text. (TIF) [file ppat.1011643.s004.TIF]
